# Supplementary material for: The Activating Effect of Strong Acid for Pd-Catalyzed Directed C–H Activation by Concerted Metalation-Deprotonation Mechanism
Source: Molecules. 2021 Jul 4;26(13):4083. doi: 10.3390/molecules26134083 (PMC8271562; doi:10.3390/molecules26134083)
Supplement: Supplementary file 1 [file molecules-26-04083-s001.zip › molecules-1272965-SI.pdf]

Supporting Information

The Activating Effect of Strong Acid for Pd-Catalyed Directed C-H ac-tivation by Concerted Metalation-Deprotonation Mechanism

Heming Jiang,[1,2] Tian-Yu Sun[2,*]

[†]Lab of Computational Chemistry & Drug Design, Key Laboratory of Chemical Genomics, Peking University Shenzhen Graduate School, Shenzhen, 518055 (China)

[§]Shenzhen Bay Laboratory, Shenzhen 518132, China.

E-mail: Tian-Yu_Sun@pku.edu.cn

Table of Contents

Computational details……………..…..……………..…...……………..…....….........S2

The scan of C-H bond of cationic species………………..…..………….....................S2

The relative free energy of different intermediates of sub2……… ……..................S2

The relative free energy barrier of intermolecular model..……..................………..S3

References……………..…..…..….......….........……....………................................ S4

Cartesian coordinates of related structure........……....……............…............. S5-S29

1. Computational details

All density functional theory (DFT) calculations were conducted with the Gaussian 09 program[1]. Geometries were optimized with M06/(LANL2DZ+f: Pd; 6-31G(d): others), single-point energies were calculated with M06/(SDD: Pd; 6-311++G(d, p): others)[2] with the SMD solvation model and dichloroethane was chosen as the solvent,[3] only singlet state isomers are discussed in this article. Vibrational frequency analysis confirmed that the structure was either a minimum or a transition state. Gibbs free energies at 298.15 K are presented for discussion.

2. The scan of C-H bond of cationic species($[PdOAc]^+$).

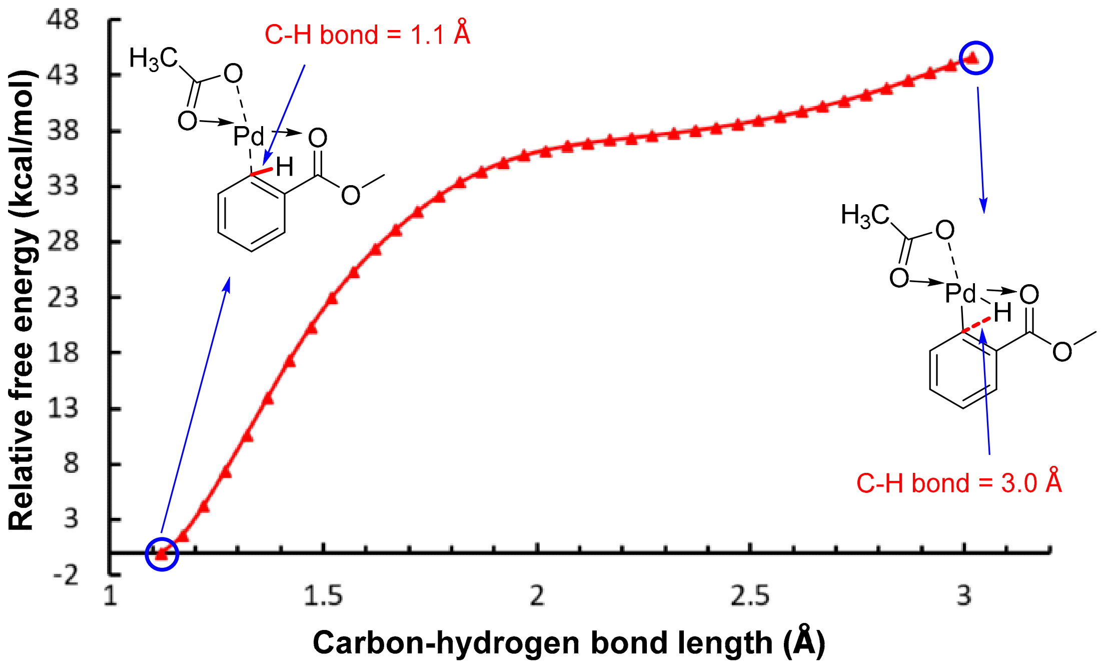

Figure S1. The scan of C-H bond of cationic species($[PdOAc]^+$).

3. The relative free energy of different intermediates of sub2

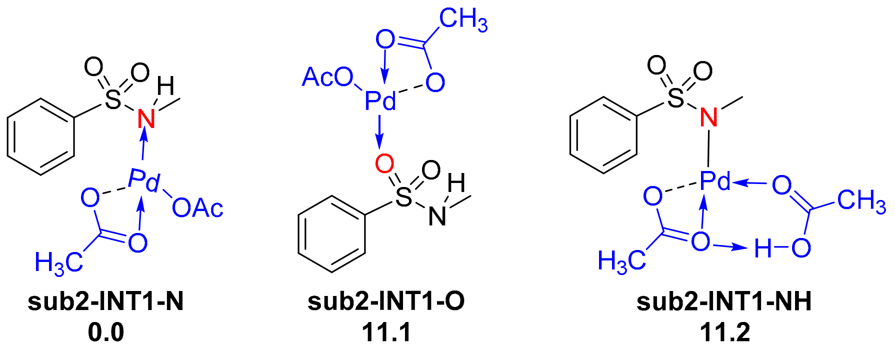

Figure S2. the relative free energy of different intermediates of sub2.

4. the relative free energy barrier of intermolecular model.

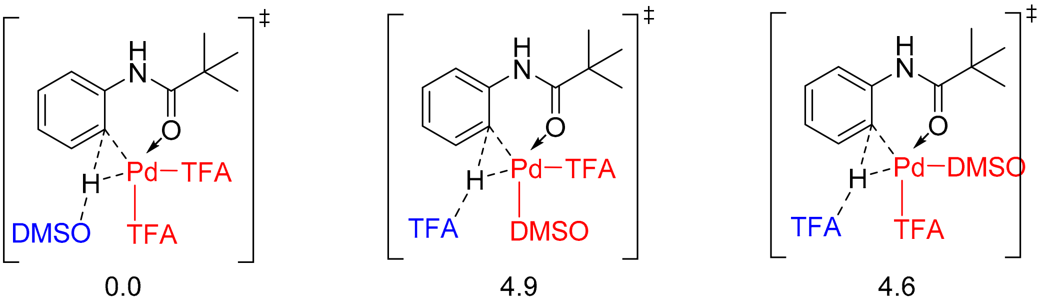

Figure S3. the relative free energy barrier of intermolecular model

REFERENCES

(1) Gaussian 09, Revision A.02, M. J. Frisch, G. W. Trucks, H. B. Schlegel, G. E. Scuseria, M. A. Robb, J. R. Cheeseman, G. Scalmani, V. Barone, B. Mennucci, G. A. Petersson, H. Nakatsuji, M. Caricato, X. Li, H. P. Hratchian, A. F. Izmaylov, J. Bloino, G. Zheng, J. L. Sonnenberg, M. Hada, M. Ehara, K. Toyota, R. Fukuda, J. Hasegawa, M. Ishida, T. Nakajima, Y. Honda, O. Kitao, H. Nakai, T. Vreven, J. A. Montgomery, Jr., J. E. Peralta, F. Ogliaro, M. Bearpark, J. J. Heyd, E. Brothers, K. N. Kudin, V. N. Staroverov, R. Kobayashi, J. Normand, K. Raghavachari, A. Rendell, J. C. Burant, S. S. Iyengar, J. Tomasi, M. Cossi, N. Rega, J. M. Millam, M. Klene, J. E. Knox, J. B. Cross, V. Bakken, C. Adamo, J. Jaramillo, R. Gomperts, R. E. Stratmann, O. Yazyev, A. J. Austin, R. Cammi, C. Pomelli, J. W. Ochterski, R. L. Martin, K. Morokuma, V. G. Zakrzewski, G. A. Voth, P. Salvador, J. J. Dannenberg, S. Dapprich, A. D. Daniels, O. Farkas, J. B. Foresman, J. V. Ortiz, J. Cioslowski, and D. J. Fox, Gaussian, Inc., Wallingford CT, 2009.
(2) (a) Hay, P. J.; Wadt, W. R. J. Chem. Phys. 1985, 82, 299. (b) Ditchfield, R.; Hehre, W. J.; Pople, J. A. J. Chem. Phys. 1971, 54, 724. (c) Hariharan, P. C.; Pople, J. A. Theor. Chim. Acta. 1973, 28, 213. (d) Zhao, Y.; Truhlar, D. G. Theor. Chem. Acc. 2008, 120, 215.
(3) Marenich, A. V. Cramer, C. J. and Truhlar, D. G. J. Phys. Chem. B, 2009, 113, 6378.

Cartesian coordinates (in Å) of related structures which calculated at the B3LYP/aug-cc-pVDZ(-pp) level of theory.

Sub1

C	-2.52783900	-0.98278200	0.34869600
C	-1.18554900	-1.30773600	0.20726200
C	-0.26097700	-0.33781700	-0.19099600
C	-0.70662600	0.95843600	-0.46043000
C	-2.04831500	1.28211900	-0.29270000
C	-2.96528100	0.31712400	0.11022900
H	-3.23519400	-1.75012200	0.65880600
H	-0.83675000	-2.31922900	0.41554100
H	-0.00633100	1.70603600	-0.82635500
H	-2.38082700	2.29756800	-0.50183400
H	-4.01594600	0.57391000	0.22959600
N	1.08461500	-0.71889700	-0.35795300
H	1.25579600	-1.66361400	-0.68812300
C	2.26335500	-0.09255900	0.00397900
O	3.32654300	-0.64181400	-0.21502200
C	2.17711300	1.25162900	0.67778800
H	2.17945200	2.05356000	-0.07139700
H	1.28274900	1.36977700	1.29887000
H	3.07711800	1.36842200	1.28776900

Sub1-INT1-OAc

C	-5.21997400	0.39580200	-1.22603000
C	-5.43754900	-0.71071300	-0.41175200
C	-4.36597800	-1.34137100	0.20524900
C	-3.06656100	-0.86816400	0.01547800
C	-2.83848800	0.24311700	-0.79952900
C	-3.92270200	0.86275700	-1.41140400
H	-6.05705900	0.89155200	-1.71344400
H	-6.44573800	-1.08948400	-0.25460900
H	-4.53548500	-2.21148200	0.84101500
H	-1.83071000	0.61581700	-0.95506100
H	-3.74123100	1.72919300	-2.04521000
N	-2.03793700	-1.53718700	0.72443800
H	-2.34949300	-2.08303100	1.51845700
C	-0.70451800	-1.43803100	0.56531400
O	-0.23220200	-0.84686400	-0.42594600
C	0.14928900	-2.07216100	1.61343300
Pd	1.59977200	0.12073300	-0.27744300
C	3.97762900	-0.37049900	-0.43722100
O	3.54835300	0.81343100	-0.24479700
O	3.11976600	-1.30547200	-0.53121400
C	-0.19219100	1.85682000	1.02910900
O	0.52765600	1.79139200	-0.05435400
C	-0.98841900	3.14001100	1.11489400
H	-0.47501100	3.96804800	0.61753000
H	-1.95440500	2.98739700	0.61491000
H	-1.18328900	3.38120000	2.16411900
C	5.43498700	-0.63812500	-0.57664500
H	6.00927100	0.07032600	0.02798300
H	5.66046700	-1.66850600	-0.28625600
H	5.72601800	-0.50384500	-1.62582900
O	-0.28092600	0.98548000	1.88887700
H	-0.36831800	-2.86755100	2.16194800
H	1.05879700	-2.47329500	1.15284300
H	0.43341200	-1.27505800	2.31272300

Sub1-INT1-TFA

C	-5.25288500	0.34099200	-1.18831600
C	-5.41485600	-0.86704700	-0.51755700
C	-4.32375400	-1.48035600	0.08275100
C	-3.06335500	-0.88679600	0.01230900
C	-2.89057200	0.32346000	-0.65931400
C	-3.99314500	0.92752500	-1.25180200
H	-6.10690900	0.82540900	-1.65713000
H	-6.39456700	-1.33694900	-0.45801500
H	-4.44751600	-2.42716700	0.60993200
H	-1.91414700	0.79093700	-0.72579900
H	-3.85531500	1.87919200	-1.76162300
N	-2.01220500	-1.53916500	0.71291000
H	-2.31123800	-2.15267800	1.46158300
C	-0.68842700	-1.38346500	0.57628200
O	-0.23360100	-0.71362900	-0.38049700
C	0.18856200	-2.03126000	1.59486400
Pd	1.59888900	0.18853800	-0.25190500
C	3.93920100	-0.38017700	-0.46625200
O	3.59810400	0.81454100	-0.25379300
O	3.06988800	-1.29168200	-0.56237700
C	-0.17547100	1.92152500	1.06907600
O	0.57022500	1.87919100	0.01843400
C	-0.97474900	3.24372500	1.09409900
C	5.41220500	-0.75938600	-0.61395000
O	-0.32856500	1.08913100	1.94418100
H	-0.31557000	-2.83548400	2.14185100

H	1.08889900	-2.42718800	1.11161700
H	0.48856800	-1.25082600	2.30687600
F	5.61183300	-1.29558800	-1.81370000
F	5.72713900	-1.66331300	0.30993200
F	6.19635100	0.29498000	-0.47347100
F	-0.16569000	4.29647900	1.01803100
F	-1.80677400	3.28300400	0.04512000
F	-1.69770200	3.34626500	2.19890300

Sub1-INT1-OTf

C	-5.83270800	-1.81578100	-1.10060300
C	-5.59887900	-2.90457100	-0.26703100
C	-4.46065100	-2.93545000	0.53022500
C	-3.56892900	-1.86793300	0.49409000
C	-3.79944900	-0.76364300	-0.32356700
C	-4.93395300	-0.75253800	-1.12565800
H	-6.72013100	-1.79327100	-1.73020300
H	-6.29874200	-3.73740300	-0.24057300
H	-4.25548500	-3.78985200	1.17493200
H	-3.10793600	0.07625600	-0.31448200
H	-5.12128100	0.10480000	-1.76902700
N	-2.43216200	-1.91039000	1.36026400
H	-2.59875300	-2.14863800	2.33064600
C	-1.18229700	-1.58257300	1.02509300
O	-0.91311800	-1.34897400	-0.18103200
C	-0.15169300	-1.53761700	2.10352900
Pd	0.76551200	-0.35281200	-0.71340500
H	-0.49002800	-2.01646900	3.02849800
H	0.76575900	-2.02715200	1.75576300
H	0.07937500	-0.48658200	2.32444200
S	-0.61760200	1.92173600	0.57606700
O	0.63024700	2.24984200	1.24590100
O	-1.63913000	1.21122700	1.34467200
C	-1.40170100	3.49348000	0.03499600
F	-1.69485600	4.20165100	1.11264400
F	-2.51296900	3.22574700	-0.63180300
F	-0.57461700	4.17621800	-0.73179100
O	-0.40358400	1.30211700	-0.81727900
S	3.36029400	-0.85060300	-0.95495600
O	2.24700500	-1.85386000	-0.70058200
O	2.62205000	0.42545500	-1.30368100
C	4.05138900	-0.52987000	0.72516600
F	3.07225800	-0.21188700	1.55417400
F	4.91622200	0.45775300	0.64679400
F	4.64904900	-1.62859000	1.14641300
O	4.45458300	-1.25630700	-1.79359100

Sub1-TS1-OAc

C	-3.12055100	1.08398100	1.86225200
C	-3.92904400	-0.00816700	1.55659800
C	-3.52465700	-0.92929600	0.59951800
C	-2.31375700	-0.75214500	-0.06715300
C	-1.50254900	0.36860700	0.17604700
C	-1.92884500	1.26135400	1.17601600
H	-3.42643500	1.79597900	2.62595400
H	-4.87304400	-0.15606800	2.07783800
H	-4.13433800	-1.80953300	0.39278900
H	-1.02415000	1.10125300	-0.81249000
H	-1.30521600	2.12911600	1.38918000
N	-1.93039700	-1.76857200	-0.98160300
H	-2.68859200	-2.26132400	-1.43852100
C	-0.71250400	-2.35792700	-1.04620100
O	0.29162900	-1.95160600	-0.44688700
C	-0.59878500	-3.58927300	-1.89248700
Pd	0.60236800	0.08557500	-0.05019400
C	0.29496900	2.73131200	-0.93250800
O	0.99455000	2.10451700	-0.07683300
O	-0.71623800	2.25885600	-1.51996600
C	2.96503900	-0.80504300	1.01595800
O	2.59026200	-0.27114100	-0.11270300
C	4.44502500	-1.11877200	1.04820100
H	4.71674000	-1.75883600	0.20092900
H	5.02320500	-0.19223800	0.95146700
H	4.70041200	-1.61472600	1.98895000
C	0.73307700	4.13008300	-1.26185300
H	1.41821300	4.08418500	-2.11726300
H	-0.12680600	4.74275500	-1.54614000
H	1.27175900	4.57404900	-0.42063300
O	2.22309700	-1.03182600	1.96398100
H	-1.54746800	-3.91277000	-2.33301100
H	0.12391200	-3.39869300	-2.69366400
H	-0.19006000	-4.39769800	-1.27679500

Sub1-TS1-TFA

C	-3.17899800	1.14642800	1.74683600
C	-3.99422100	0.07104400	1.40100900
C	-3.55693400	-0.87334700	0.48167000
C	-2.30216700	-0.73825600	-0.10798400
C	-1.48231400	0.37180600	0.16702000
C	-1.94359400	1.28613500	1.13459100
H	-3.51341200	1.87532900	2.48151600

H	-4.97212900	-0.04628300	1.86383500
H	-4.17591600	-1.74034100	0.24980100
H	-1.01985400	1.12192900	-0.83619000
H	-1.31717600	2.14434100	1.37721000
N	-1.88418500	-1.78057700	-0.97291500
H	-2.62277100	-2.28652000	-1.44820500
C	-0.66535600	-2.36804500	-0.97082800
O	0.31271000	-1.93548000	-0.34165200
C	-0.50708500	-3.62639100	-1.76407800
Pd	0.60729700	0.08443000	0.00113500
C	0.30220400	2.70939900	-0.90125200
O	1.00070200	2.11948400	-0.04217900
O	-0.69051200	2.25660200	-1.52151700
C	2.98445500	-0.84459900	1.03843900
O	2.60914900	-0.28116700	-0.05677500
C	4.48028200	-1.21614400	0.97950600
C	0.64866100	4.17877100	-1.19194800
O	2.32050000	-1.10074000	2.02588700
H	-1.43431700	-3.97265900	-2.23138300
H	0.24912700	-3.45972700	-2.53934700
H	-0.11884300	-4.40775400	-1.10178600
F	0.70611400	4.38084900	-2.50240100
F	-0.32053200	4.94373600	-0.68662500
F	1.79848900	4.53721100	-0.65092000
F	5.23050800	-0.14295000	0.72410900
F	4.69530700	-2.10749600	0.00506900
F	4.89519300	-1.74894500	2.12161500

Sub1-TS1-OTf

C	2.78656300	1.35289700	2.60935600
C	3.11859700	2.68602100	2.37096000
C	2.60085700	3.36013100	1.27277400
C	1.74499000	2.69893500	0.39525700
C	1.45350900	1.32677200	0.55962200
C	1.96158200	0.69144800	1.71646300
H	3.18048500	0.83542100	3.48071700
H	3.77465400	3.21604600	3.05877300
H	2.82796100	4.41542000	1.12108100
H	1.84423400	0.52711500	-0.43683200
H	1.72472000	-0.36096400	1.87169000
N	1.18812500	3.46125300	-0.65285500
H	1.70663200	4.29012400	-0.92149700
C	-0.07409000	3.36750200	-1.13548500
O	-0.87677800	2.47882600	-0.81415400
C	-0.52265800	4.41870300	-2.09897600
Pd	-0.32716400	0.58957600	-0.22245300
H	0.20608100	5.22212600	-2.24485300
H	-0.73455700	3.94284700	-3.06327300
H	-1.46637300	4.84037400	-1.73700600
S	1.53684600	-1.72382400	-0.90674600
O	2.33243200	-0.46566700	-1.18854100
O	0.37850600	-1.39150800	-0.00212100
C	0.75968300	-2.08969000	-2.53641700
F	0.09108000	-1.02983200	-2.95622800
F	-0.05620000	-3.11181000	-2.39668300
F	1.71309400	-2.38346200	-3.40113200
O	2.29770000	-2.88775500	-0.52385100
S	-3.30859900	0.19929600	0.06831900
O	-2.91759300	1.16666400	1.08956600
O	-2.16067100	-0.08727600	-0.90488600
C	-3.40561800	-1.40308200	0.95914000
F	-2.25606400	-1.63693700	1.58443600
F	-3.64475000	-2.39027500	0.11192500
F	-4.37895400	-1.35148500	1.85619500
O	-4.57344700	0.34229000	-0.62526800

Sub2

C	2.80912700	0.95778400	-0.40780200
C	1.44276000	1.16586800	-0.24298800
C	0.63844100	0.08529600	0.09825200
C	1.16237400	-1.19110100	0.28834700
C	2.52627900	-1.38580400	0.11988400
C	3.34595900	-0.31279900	-0.22824000
H	3.45533900	1.79212800	-0.67339600
H	0.99437600	2.14949000	-0.36610200
H	0.50185200	-2.01101300	0.56426900
H	2.95464600	-2.37585000	0.26318200
H	4.41535900	-0.47074400	-0.35752500
S	-1.10235300	0.33913500	0.33731000
O	-1.54610900	-0.36629100	1.52968900
O	-1.37505000	1.76434000	0.17699800
N	-1.71282400	-0.53968600	-0.96820100
C	-3.14408800	-0.83540100	-0.85862400
H	-3.43164800	-1.43667300	-1.72659400
H	-3.30774900	-1.42725600	0.04594000
H	-3.77479600	0.06508700	-0.81537500
H	-1.49580600	-0.02097400	-1.82042000

Sub2-INT1-OAc-N

Pd	0.66804600	0.78055700	0.25050000
C	-2.55462000	0.31666400	-1.93971300

C	-1.53305300	-0.52489300	-1.51826300
C	-1.70306100	-1.23186100	-0.32846000
C	-2.86296500	-1.13625100	0.43266300
C	-3.87733800	-0.29106300	-0.00483700
C	-3.71846500	0.43547900	-1.18111300
H	-2.44344200	0.87732900	-2.86543200
H	-0.61607500	-0.63926800	-2.09526600
H	-2.96683400	-1.72550800	1.34165200
H	-4.79438300	-0.20262500	0.57378600
H	-4.51584300	1.09548600	-1.51868200
O	0.47624500	-2.63345000	-0.85006900
C	-0.61671000	2.84906700	0.45355200
O	-0.88906700	1.82846400	1.17068200
C	3.11620400	-0.57415500	-0.57933200
O	3.17279600	-1.17835600	0.49672300
O	2.17973600	0.23876200	-0.95286400
C	4.17863500	-0.77277500	-1.63060200
H	3.83620400	-1.55172700	-2.32333300
H	4.34686400	0.14215100	-2.20587800
H	5.10501000	-1.11238100	-1.15895200
C	-1.42864100	4.09144600	0.57246300
H	-2.47903000	3.86314600	0.35745500
H	-1.38020200	4.46514100	1.60168300
H	-1.06361100	4.85647000	-0.11788300
O	0.35679500	2.76793600	-0.35735100
N	0.58617300	-1.09208500	1.17403000
H	1.60260800	-1.36452400	1.06136300
C	0.18696900	-0.95513300	2.58432500
H	0.93647000	-0.32698900	3.07579400
H	-0.78618900	-0.45515000	2.64641900
H	0.13953400	-1.93463700	3.07227600
S	-0.37166200	-2.24467700	0.25904200
O	-0.89849300	-3.20812100	1.21100800

Sub2-INT1-TFA-N

Pd	0.64756600	0.71760900	0.27802000
C	-2.37849400	0.31738400	-1.98439200
C	-1.44252300	-0.60105600	-1.52615600
C	-1.67023100	-1.22244400	-0.29733900
C	-2.80852200	-0.97526400	0.46423000
C	-3.73432600	-0.05313000	-0.01105200
C	-3.51248400	0.59601200	-1.22252800
H	-2.22009200	0.81822000	-2.93686500
H	-0.54541200	-0.83228100	-2.09928200
H	-2.96104000	-1.49890000	1.40602100
H	-4.62869000	0.16260200	0.56903800
H	-4.23454900	1.32751900	-1.58038100
O	0.45035800	-2.75071600	-0.70630200
C	-0.70564500	2.73291000	0.45301000
O	-0.90222800	1.76301800	1.24384400
C	3.06277300	-0.63231100	-0.62259700
O	3.20585300	-1.26483000	0.41409600
O	2.11712800	0.16354800	-0.97064500
C	4.12660100	-0.72640000	-1.73848900
C	-1.65695000	3.92596400	0.54247000
O	0.21957100	2.70468300	-0.39541900
N	0.60566400	-1.11579000	1.24459500
H	1.59529200	-1.43801400	1.12253500
C	0.24330600	-0.94810100	2.66296100
H	1.00939100	-0.32031500	3.12863300
H	-0.72492900	-0.44215200	2.73943700
H	0.19839600	-1.92067700	3.16456900
S	-0.41173800	-2.27995300	0.35709000
O	-0.98623300	-3.15939700	1.35802900
F	4.68079900	0.46975600	-1.93575700
F	3.56508800	-1.12016400	-2.87966800
F	5.08074100	-1.58504200	-1.41811900
F	-1.27238900	4.91140100	-0.24865100
F	-1.71267200	4.36523900	1.79466100
F	-2.87927300	3.53224000	0.18055700

Sub2-INT1-OTf-N

Pd	0.23096300	0.76781700	0.02549600
C	-2.54505200	-0.23972900	-2.31234800
C	-1.53304000	-0.98953200	-1.72668500
C	-1.75783300	-1.52538200	-0.45714500
C	-2.96007500	-1.35827700	0.22419100
C	-3.96163600	-0.60802900	-0.38108100
C	-3.74834300	-0.04318600	-1.63564900
H	-2.39164400	0.19760400	-3.29619200
H	-0.58205400	-1.15533600	-2.23191800
H	-3.10056300	-1.80862600	1.20491200
H	-4.90814500	-0.45635400	0.13258500
H	-4.52912100	0.56071700	-2.09336500
O	0.55090300	-2.81949800	-0.59846500
O	-1.49928000	1.62561800	0.89914200
O	3.13018500	-0.54982400	0.86325400
O	1.82806900	0.21589300	-1.10071500
C	4.09078200	-0.88817700	-1.58265600
C	-0.75857300	4.15896300	1.15412000

O	-0.31889000	2.58919700	-0.94567600
N	0.41079000	-0.95687600	1.14116200
H	1.42948300	-1.17109600	1.08108000
C	0.01922900	-0.71269100	2.54125500
H	0.70000000	0.04564400	2.94257900
H	-1.00885700	-0.33759300	2.57688000
H	0.09972900	-1.63545100	3.12564800
S	-0.42286500	-2.34272100	0.35919300
O	-0.93415100	-3.15775800	1.44476000
F	4.09995700	-0.33032300	-2.77888200
F	3.49920200	-2.06563100	-1.64156900
F	5.33088100	-1.03494500	-1.14984500
F	-0.55985900	5.26135300	0.46316100
F	0.38043800	3.73595900	1.67031000
F	-1.63315000	4.37526100	2.11776600
O	3.87405000	1.48176000	-0.39981200
O	-2.68448700	3.37068200	-0.49203200
S	-1.43862900	2.86951900	0.02682200
S	3.19919700	0.20192100	-0.39821500

Sub2-TS1-OAc-N

C	3.54986300	0.79897600	1.90261900
C	3.28994900	-0.22622700	0.99625300
C	2.17433800	-0.11634200	0.18168900
C	1.27374800	0.95894100	0.23005700
C	1.54925700	1.94801300	1.18445000
C	2.67635800	1.87681400	1.99890800
H	4.42498100	0.74247300	2.54653700
H	3.92494900	-1.10781100	0.93336600
H	0.63186300	1.58385900	-0.75089000
H	0.87774200	2.80032800	1.27249500
H	2.86921600	2.66714300	2.72198100
S	1.79614900	-1.46861300	-0.89453300
O	1.75375300	-1.04051400	-2.27936200
O	2.53721900	-2.65183400	-0.49264100
N	0.11166400	-1.68572900	-0.43862700
C	-0.59805700	-2.47743000	-1.46902100
H	-0.08987800	-3.43768800	-1.62544300
H	-1.61382700	-2.64876100	-1.11172800
H	-0.62168400	-1.91058600	-2.40247700
Pd	-0.73626000	0.19468300	-0.05289900
C	-2.66536000	-1.56314100	1.03468600
O	-2.59157500	-0.61076800	0.15342800
C	-1.05828100	2.87827900	-0.76451800
O	0.03731700	2.68936900	-1.35839100
O	-1.59457800	2.05825100	0.04861700
C	-1.81446300	4.14803600	-1.03092800
H	-2.43447900	4.41717100	-0.17176800
H	-1.12430800	4.95591300	-1.28838800
H	-2.47790300	3.97945700	-1.88809300
C	-4.08863600	-1.90091800	1.41274400
H	-4.12229900	-2.86452900	1.92841800
H	-4.47118800	-1.12281300	2.08450600
H	-4.73556800	-1.91244600	0.52952800
O	-1.70966500	-2.15605900	1.53988500
H	0.00697500	-2.14805000	0.48152100

Sub2-TS1-TFA-N

C	3.60964700	0.89372600	1.68137700
C	3.30832000	-0.16690000	0.82813400
C	2.13713400	-0.09969400	0.09325200
C	1.22336400	0.96246600	0.17204300
C	1.54226600	1.98882700	1.07364300
C	2.72663300	1.96091000	1.80620800
H	4.52856200	0.87333200	2.26324900
H	3.95571400	-1.03803500	0.74822900
H	0.57544000	1.57810900	-0.83695800
H	0.86837600	2.83863200	1.18117100
H	2.95689600	2.77756000	2.48738100
S	1.68748500	-1.48526300	-0.90925300
O	1.51234000	-1.10270800	-2.29515400
O	2.45915900	-2.65420800	-0.52972700
N	0.03975600	-1.69155200	-0.29222200
C	-0.72810400	-2.57852200	-1.20226200
H	-0.18283700	-3.51892800	-1.35199100
H	-1.69784000	-2.78818300	-0.74856900
H	-0.86807000	-2.06963800	-2.15851200
Pd	-0.78442200	0.20964300	0.00482600
C	-2.70627000	-1.48251100	1.13022000
O	-2.65574900	-0.58797900	0.21192900
C	-1.04019500	2.87891500	-0.76054100
O	-0.00422000	2.65773300	-1.43145600
O	-1.60150800	2.11060100	0.05990300
C	-1.61526200	4.30058500	-0.84976300
C	-4.10706100	-2.11743600	1.22312100
O	-1.79852200	-1.93288300	1.81615800
H	0.06100900	-2.10203800	0.65331600
F	-2.89116300	4.34238500	-0.51190000
F	-1.47126200	4.80228700	-2.06497800
F	-0.91685700	5.05104700	0.00993700

F	-4.21668300	-2.99133300	0.20831100
F	-5.08322500	-1.22643800	1.10888800
F	-4.26912800	-2.78021200	2.35786900

Sub2-TS1-OTf-N

C	3.49963800	0.87273800	1.73912900
C	3.25403500	-0.15393400	0.82451900
C	2.08072500	-0.10439500	0.09736200
C	1.11538200	0.90827900	0.23761800
C	1.36928100	1.88631400	1.21343400
C	2.56038500	1.87936000	1.93596100
H	4.42017700	0.86580200	2.31892600
H	3.94570900	-0.98475200	0.69887800
H	0.54935200	1.57609400	-0.78906900
H	0.63458500	2.67101200	1.38292300
H	2.74821500	2.65862700	2.67149000
S	1.67439300	-1.44720900	-0.97864000
O	1.51229300	-0.99061300	-2.34244900
O	2.45645100	-2.62120900	-0.64380100
N	0.01800500	-1.71029200	-0.39198500
C	-0.75842700	-2.48724700	-1.39282200
H	-0.23382600	-3.42540900	-1.61392900
H	-1.73773600	-2.69836900	-0.96262400
H	-0.86441600	-1.89219800	-2.30285500
Pd	-0.83964300	0.12154800	0.05431300
O	-2.69616200	-0.79909100	0.28799600
O	0.00825700	2.53846200	-1.53878700
O	-1.65007500	2.06623000	0.21242100
C	-1.33395000	4.55301300	-0.48639900
C	-4.16148300	-2.69187800	1.21016200
O	-3.15490300	-0.78215800	2.74950200
H	0.01849700	-2.22602300	0.50494700
F	-0.37101500	4.67397700	0.41578500
F	-2.49102900	4.87106700	0.05183400
F	-1.07639400	5.33608200	-1.51313200
F	-3.86334300	-3.42642500	0.14324600
F	-5.24793400	-1.98644300	0.96578700
F	-4.37131900	-3.49257300	2.23995600
O	-1.59342200	-2.46696000	1.74798200
O	-2.42688900	2.74019400	-2.09672500
S	-2.75518000	-1.57731200	1.60659800
S	-1.40872800	2.81129500	-1.07905500

Sub2-INT1-OAc-O

C	-4.42533100	1.18516600	-0.01051300
C	-3.10492600	0.78037100	0.12995000
C	-2.78922200	-0.54579100	-0.15969900
C	-3.74077600	-1.46480400	-0.58276600
C	-5.05782200	-1.03800800	-0.72342800
C	-5.39617700	0.28050400	-0.43839400
H	-4.69965200	2.21372800	0.21486500
H	-2.33324200	1.47097400	0.47373100
H	-3.44529800	-2.48957800	-0.79708000
H	-5.81970900	-1.73966600	-1.05668200
H	-6.42816600	0.60899800	-0.54834300
S	-1.11264300	-1.08970800	0.00203400
O	-1.02011800	-2.51855900	-0.23633900
O	-0.36105900	-0.15843300	-0.90944800
N	-0.70050300	-0.76241700	1.55605800
C	0.11971800	-1.74751600	2.26359000
H	1.12955800	-1.85209100	1.84166300
H	0.19013100	-1.41574800	3.30333100
H	-0.37656800	-2.72115000	2.23843900
Pd	1.64847700	0.27097400	-0.43684400
C	0.32514200	2.65230200	0.33933700
O	1.16014100	2.20198100	-0.53555100
C	3.72976500	-0.92454400	-0.07242700
O	2.64620600	-1.55808000	-0.28656700
O	3.66843600	0.34860900	-0.03091700
C	5.02546700	-1.63353000	0.09371800
H	5.69015100	-1.06044700	0.74715400
H	4.85757900	-2.63820700	0.49287400
H	5.50925500	-1.72959200	-0.88618600
C	-0.03850900	4.10127200	0.12165100
H	-0.29339100	4.56594700	1.07875500
H	0.76928500	4.64898200	-0.37150900
H	-0.92272400	4.14449800	-0.52774200
O	-0.18685700	2.01119300	1.26411800
H	-0.40700500	0.22884700	1.63334800

Sub2-INT1-OAc-NH

Pd	0.88730100	0.40396300	-0.76480400
C	-3.74776800	0.95512600	-0.05635500
C	-2.97260600	-0.09038300	-0.54084000
C	-1.94672500	-0.59037600	0.25475500
C	-1.68982300	-0.08126400	1.52323700
C	-2.47204600	0.96737700	1.99780900
C	-3.49553700	1.48382100	1.20795100
H	-4.55131100	1.36148800	-0.66744200
H	-3.14513900	-0.51450800	-1.52804900
H	-0.88749800	-0.51132000	2.12046900

H	-2.28764200	1.37479800	2.99043000
H	-4.10753900	2.30266900	1.58312000
O	-1.79477800	-2.91375100	-1.01500900
N	-0.14176500	-1.09213800	-1.61308100
C	2.58731500	-0.93150600	1.12026100
O	2.36206700	-0.93804900	-0.09560500
C	0.36142700	2.77794800	-0.28205500
O	-0.38354200	2.08892200	-1.03536900
O	1.43590800	2.25523500	0.18423200
C	0.01632000	4.18885300	0.05283600
H	0.12764900	4.35112200	1.13052700
H	0.71543700	4.86462600	-0.45430500
H	-1.00450500	4.41925400	-0.26346000
C	3.14117100	-2.12960600	1.79853400
H	3.59722500	-1.88090200	2.75940900
H	2.29364500	-2.81064400	1.95750500
H	3.85274200	-2.63423800	1.13965700
O	2.33198900	0.08376700	1.91451700
H	1.98093300	0.85974200	1.40900000
C	0.67822400	-1.99780300	-2.41619600
H	1.47235300	-2.49892200	-1.84220100
H	0.02800600	-2.76214200	-2.86400100
H	1.12861300	-1.41336600	-3.22568700
S	-0.94049700	-1.91212900	-0.38412600
O	-0.04267200	-2.34193200	0.69790600

Sub3

C	-2.33863900	0.85805500	0.49445300
C	-0.99263800	1.18421500	0.40031000
C	-0.07428800	0.25573800	-0.09413400
C	-0.52877900	-0.98922200	-0.53456700
C	-1.88044200	-1.30821400	-0.45950100
C	-2.78440000	-0.38996400	0.06544200
H	-3.04640700	1.58263700	0.89355700
H	-0.62688500	2.16477700	0.69901900
H	0.17166500	-1.70218900	-0.96793800
H	-2.22943600	-2.27549100	-0.81726100
H	-3.84140200	-0.64332900	0.13092500
C	1.34716200	0.70020300	-0.22037200
O	1.62310800	1.85928500	-0.49080700
N	2.33719600	-0.23562500	-0.05356000
H	3.25256400	0.17931500	-0.18689100
C	2.28069500	-1.48851300	0.66928100
H	3.10649400	-1.54155400	1.38916500
H	1.34434000	-1.56124500	1.23097600
H	2.35180400	-2.36161300	0.00435000

Sub3-INT1-OAc

C	-5.52900200	-1.37744700	0.33745800
C	-4.49527500	-1.70388400	1.21960800
C	-3.23158100	-1.14185900	1.05470200
C	-2.99949900	-0.22441200	0.01879000
C	-4.03675600	0.09281400	-0.87042200
C	-5.29559500	-0.48707100	-0.71234400
H	-6.51183200	-1.82344400	0.46246300
H	-4.67173800	-2.40412100	2.03095200
H	-2.41475200	-1.40665600	1.71773400
H	-3.85535300	0.76437100	-1.70373400
H	-6.09046800	-0.24748100	-1.41290200
C	-1.61763300	0.31433400	-0.14841100
O	-0.67057900	-0.50172300	0.04603600
N	-1.39338200	1.58516800	-0.49325200
C	-2.33914300	2.69359300	-0.51779300
H	-1.85096800	3.57306100	-0.08740800
H	-2.65022900	2.93963400	-1.53997700
H	-3.22468300	2.46261100	0.07594400
Pd	1.38078100	-0.32058500	-0.16166000
C	3.13547800	-2.01353400	-0.21980600
O	1.91283200	-2.34916400	-0.06459500
O	3.38859100	-0.76802100	-0.37411500
C	1.94303200	2.35254000	0.59339600
O	1.36013300	1.69356900	-0.40397400
O	2.31013000	1.84446800	1.64005900
C	2.12860600	3.83243100	0.28256600
H	2.49070500	4.35241100	1.17127000
H	2.85632900	3.94586400	-0.52851800
H	1.19154200	4.28632000	-0.05813000
C	4.23902600	-3.02736800	-0.18931400
H	3.86269900	-4.00307600	-0.50543000
H	5.06375000	-2.70485400	-0.82961900
H	4.61555500	-3.11496000	0.83672200
H	-0.39161500	1.81722300	-0.57811100

Sub3-INT1-TFA

C	-5.43696900	-1.33256500	0.38785700
C	-4.35622000	-1.72747500	1.17209700
C	-3.10225700	-1.17313700	0.95657100
C	-2.93152900	-0.19549500	-0.02762000
C	-4.01573000	0.19102900	-0.82052000
C	-5.26424700	-0.38221900	-0.61436800
H	-6.41784100	-1.77492900	0.55228100

H	-4.48944700	-2.47785900	1.94859200
H	-2.24330400	-1.48868000	1.54476300
H	-3.87715700	0.91586700	-1.62112400
H	-6.10360200	-0.08963000	-1.24195100
C	-1.56806300	0.33996800	-0.25147100
O	-0.62712300	-0.49742800	-0.16658300
N	-1.36541100	1.62382900	-0.53555300
C	-2.30560200	2.72410600	-0.41160900
H	-1.77956700	3.57449300	0.03231000
H	-2.69948300	3.03186700	-1.38836400
H	-3.13978600	2.44715200	0.23851200
Pd	1.41067100	-0.34644100	-0.25396500
C	3.12186900	-2.03766000	-0.13474200
O	1.91105200	-2.38479600	-0.06370500
O	3.43335700	-0.82275500	-0.28256900
C	1.96029400	2.30270800	0.46085300
O	1.42155600	1.65031900	-0.52927500
O	2.42436500	1.87223200	1.48927400
C	1.91180100	3.81572000	0.16775800
C	4.20182400	-3.11401500	-0.03903200
H	-0.38393000	1.87140900	-0.70125900
F	2.37886200	4.52530000	1.17835200
F	0.63262000	4.18751900	-0.04570600
F	2.60391800	4.11722800	-0.92637500
F	5.41121900	-2.58944400	-0.13603700
F	4.09229500	-3.74746100	1.12426700
F	4.03026500	-3.99737800	-1.01840300

Sub3-INT1-OTf

C	1.18364700	-1.28750300	-0.89911200
O	0.44116900	-0.43186100	-1.45339100
Pd	-1.06078400	0.52180600	-0.46199100
C	0.80536600	-1.95279300	0.36445900
C	-0.48253400	-2.48574800	0.47497900
C	1.67016900	-1.96795400	1.46062200
C	-0.89108600	-3.05509700	1.67375300
H	-1.15455600	-2.46202200	-0.38341800
C	1.24669100	-2.52114500	2.66215600
H	2.65115800	-1.50198800	1.38697100
C	-0.02840600	-3.06924800	2.76667800
H	-1.89067800	-3.47636100	1.75736700
H	1.91046900	-2.51192500	3.52391800
H	-0.35621000	-3.50206200	3.70986400
S	-3.69591400	0.73779500	-0.82803300
O	-2.69482900	0.00131800	-1.69757900
O	-4.74393300	1.47573000	-1.47944100
C	-4.52211900	-0.60379500	0.12753500
F	-5.20722300	-1.35893900	-0.70970200
F	-5.32989100	-0.06145000	1.01469600
F	-3.61282900	-1.34298200	0.74517800
O	-2.84040400	1.45187500	0.19523400
S	1.07043400	2.36976200	0.71595100
O	1.03155900	2.75621800	-0.68451800
O	0.22810800	1.10379900	0.98227200
C	2.74689800	1.66414300	0.98926700
F	2.99468300	0.69348500	0.10025800
F	2.84617600	1.14544300	2.20369300
F	3.65514200	2.61010000	0.83871600
O	0.91478800	3.35241500	1.76738500
C	3.30481100	-2.59527800	-1.16777600
H	2.84680800	-3.35638600	-0.53098000
H	3.67580100	-3.07767600	-2.07741400
H	4.15142500	-2.14441200	-0.63344500
N	2.31986900	-1.59482400	-1.52616900
H	2.50934200	-1.00021900	-2.32821100

Sub3-TS1-OAc

C	-3.22593700	-2.36700100	-0.71506500
C	-1.88410700	-2.15020600	-0.41327700
C	-1.41765900	-0.89519800	-0.01136800
C	-2.35708300	0.16169500	0.05114700
C	-3.69862700	-0.05169500	-0.26100600
C	-4.13607300	-1.31958600	-0.63048500
H	-3.56161600	-3.35614600	-1.02162000
H	-1.17969900	-2.98007800	-0.45659800
H	-0.41112500	-1.20702700	0.80847200
H	-4.40217500	0.78115400	-0.27594700
H	-5.18412100	-1.48158300	-0.87467200
C	-1.82626400	1.52538800	0.25234900
O	-0.64694000	1.79523700	-0.07175100
N	-2.61931200	2.49117700	0.73770200
C	-2.12694700	3.84235700	0.93655000
H	-1.67163900	4.20856400	0.01132300
H	-2.96574200	4.48789800	1.20731300
H	-1.36765800	3.87480100	1.72739200
Pd	0.48873600	0.06335700	-0.43027400
C	3.02419900	1.13424000	-0.08580000
O	2.09950700	1.11137900	-1.00730900
O	2.94176000	0.62785300	1.02508500
C	1.45936100	-2.23173500	0.70247600

O	1.61539200	-1.63269700	-0.41071700
O	0.48978200	-2.04726800	1.48624200
C	2.54202100	-3.19338700	1.08804400
H	2.18995400	-3.89823000	1.84520400
H	2.92781700	-3.71692600	0.20864700
H	3.36084300	-2.59448100	1.50736900
C	4.26125800	1.87622000	-0.54835600
H	3.99394200	2.87297000	-0.91689700
H	4.97496100	1.95957800	0.27615300
H	4.72445000	1.33687000	-1.38310900
H	-3.51054600	2.22378600	1.13179300

Sub3-TS1-TFA

C	-3.08602200	-2.40941900	-0.68897600
C	-1.74956200	-2.14888700	-0.39565400
C	-1.32626900	-0.87549400	-0.00190000
C	-2.29831300	0.15410200	0.05161700
C	-3.63164100	-0.10450900	-0.25461000
C	-4.02800900	-1.39079900	-0.60954300
H	-3.39072900	-3.41140200	-0.98468400
H	-1.02550900	-2.96274700	-0.43411400
H	-0.33587600	-1.17990800	0.88013800
H	-4.36331500	0.70334700	-0.27580000
H	-5.07104400	-1.58932000	-0.84712100
C	-1.79595500	1.52934700	0.23190500
O	-0.60676900	1.80080900	-0.07062700
N	-2.60128900	2.50213600	0.66771800
C	-2.12388000	3.86601200	0.82801100
H	-1.66782600	4.21037100	-0.10517000
H	-2.97126900	4.50836300	1.07692600
H	-1.36990800	3.92652100	1.62163900
Pd	0.54615800	0.10804000	-0.38793500
C	2.99943100	1.29673300	0.02494000
O	2.14899600	1.20773600	-0.94019200
O	2.93309000	0.83076700	1.14653300
C	1.46173300	-2.23062900	0.70822900
O	1.70815900	-1.58847300	-0.34253900
O	0.52954300	-2.03211400	1.52377300
C	2.31437700	-3.48164800	0.96079800
C	4.21886100	2.13821600	-0.40659200
H	-3.51055200	2.24735000	1.02812500
F	4.81022600	1.60226600	-1.47553400
F	5.11721200	2.22296000	0.56539800
F	3.83604200	3.37827300	-0.72726600
F	3.44436000	-3.46410200	0.27839800
F	1.59022300	-4.53558500	0.56418500
F	2.58633100	-3.61905300	2.24858200

Sub3-TS1-OTf

Pd	-0.23440400	-0.17274900	-0.10281000
C	-2.18158500	0.20513600	0.59820000
C	-3.70674000	0.42516100	2.47640400
C	-4.52336800	-0.56628600	1.94555200
C	-4.16600600	-1.20853700	0.76214100
C	-3.01169600	-0.82752500	0.08763600
H	-3.97453000	0.90729100	3.41428000
H	-5.42997200	-0.86440400	2.46788900
H	-4.77102500	-2.04331600	0.40841700
C	-2.46377000	-1.58816600	-1.04940600
C	-2.53678500	0.79393200	1.81690500
H	-1.90691100	1.57520300	2.24108300
O	-1.23422300	-1.50491200	-1.30681800
C	-2.66886300	-3.20110800	-2.85941200
H	-1.77621700	-3.72022100	-2.49792100
H	-3.41526000	-3.93320000	-3.17478600
H	-2.38394200	-2.57505700	-3.71313900
H	-1.68111200	1.29409800	-0.08935700
S	0.05700200	2.80014300	0.30161800
O	0.60564000	1.50926900	0.85315400
O	0.11840200	3.92921100	1.19750000
C	1.13737500	3.17200300	-1.14295800
F	2.36386900	3.37118700	-0.70940000
F	0.68548300	4.25705700	-1.74249400
F	1.11179400	2.15242600	-1.98021200
O	-1.29094700	2.53732700	-0.34480600
S	2.02910900	-2.15774400	-0.15208700
O	2.94232500	-2.86045900	-1.03025600
O	0.89922700	-2.85927700	0.44935200
C	3.03756100	-1.58517800	1.27176800
F	3.50467100	-2.63498900	1.93010700
F	2.28089300	-0.86289900	2.09145900
F	4.04950700	-0.84544800	0.85215800
O	1.60217900	-0.81069500	-0.75331600
N	-3.22867500	-2.39464700	-1.78533200
H	-4.22983300	-2.37153400	-1.64927300

Sub4

C	1.95523200	-1.13691700	-0.00005200
C	0.57071700	-1.21687900	-0.00001000
C	-0.20612300	-0.05415900	0.00000600
C	0.42809200	1.19123300	-0.00002000

C	1.81588600	1.27244200	-0.00006200
C	2.57960000	0.10914300	-0.00007800
H	2.55352900	-2.04646600	-0.00006400
H	0.05526800	-2.17539400	0.00001000
H	-0.16013900	2.10788600	-0.00000800
H	2.30312800	2.24592900	-0.00008300
H	3.66667700	0.17319400	-0.00011100
C	-1.69102200	-0.20302100	0.00005100
O	-2.20735200	-1.30508800	0.00007300
C	-2.53688700	1.04630800	0.00007300
H	-2.33071500	1.66474800	0.88385200
H	-2.33077800	1.66473400	-0.88373000
H	-3.59111800	0.75717700	0.00011200

Sub4-INT1-OAc

C	5.37695800	-0.06046200	-0.78844200
C	4.26903400	0.08072200	-1.62164700
C	2.99002200	-0.06291900	-1.10596600
C	2.81512200	-0.33191000	0.25995600
C	3.93186500	-0.45583900	1.09531500
C	5.20930000	-0.33255200	0.56732400
H	6.38021600	0.04757600	-1.19747700
H	4.40454800	0.30721300	-2.67718900
H	2.10632600	0.06504100	-1.72932600
H	3.80850400	-0.67772200	2.15395100
H	6.07713300	-0.44555900	1.21392500
C	1.43708800	-0.45902800	0.75405200
O	0.56441800	-0.76171200	-0.07416200
Pd	-1.45520600	-0.37113400	0.12681100
C	-3.56632500	-1.57301800	0.01511100
O	-2.46470200	-2.20208900	-0.05809900
O	-3.51810400	-0.30919100	0.17947000
C	-0.42770800	2.13861900	-0.59856900
O	-0.95487700	1.54089900	0.44337200
O	-0.15559100	1.60115100	-1.66042400
C	-0.19927000	3.61282000	-0.34543500
H	0.41138500	4.03616900	-1.14771400
H	-1.16646200	4.12887900	-0.31760100
H	0.28162900	3.77647400	0.62537300
C	-4.87289900	-2.27282400	-0.11522500
H	-4.76612400	-3.32811100	0.15215700
H	-5.62579000	-1.78728900	0.51336800
H	-5.21114100	-2.20999000	-1.15689200
C	1.07300400	-0.17074400	2.17117900
H	0.54060800	0.79172200	2.17277600
H	0.36088300	-0.92547400	2.52799000
H	1.92784500	-0.10537700	2.84846300

Sub4-INT1-TFA

C	-1.42152300	0.92860100	-0.69526900
O	-0.46715400	0.35632300	-1.25150200
Pd	1.24473500	-0.25419800	-0.26854200
C	3.64486900	-0.12974400	-0.51981400
O	2.90988200	0.50666700	-1.32392500
O	3.13775200	-0.81427000	0.41117400
C	-0.70894400	-2.03794300	0.61137300
O	-0.02544600	-1.03359200	1.06075100
O	-0.58935100	-2.64476900	-0.42948000
C	-1.87861400	-2.34794000	1.56860700
C	5.16123700	-0.04296800	-0.68713000
C	-1.26098700	1.77976800	0.48843600
C	-0.06311000	2.48263000	0.67737300
C	-2.29709700	1.90651800	1.42070700
C	0.09307700	3.29893200	1.78697100
H	0.72966400	2.41319600	-0.06802800
C	-2.12397800	2.70272800	2.54376100
H	-3.22194900	1.34780000	1.28926200
C	-0.93269100	3.40067600	2.72456700
H	1.01640200	3.85779400	1.92281500
H	-2.91952100	2.77858400	3.28172900
H	-0.80318900	4.03186200	3.60179100
F	5.54430200	1.22246000	-0.53111900
F	5.78630400	-0.79569800	0.20150200
F	5.49812700	-0.43780700	-1.90954400
F	-1.52119600	-2.33699100	2.84614100
F	-2.81717500	-1.38781500	1.39996700
F	-2.44029700	-3.51216300	1.29310800
C	-2.77458800	0.70039900	-1.28077300
H	-3.41045400	1.59030300	-1.23946600
H	-2.67510000	0.33314800	-2.30563800
H	-3.25345900	-0.09377600	-0.68672100

Sub4-INT1-OTf

C	1.42997400	-1.21624900	-0.60709900
O	0.69528600	-0.43896300	-1.24046800
Pd	-0.98175300	0.47079900	-0.47800400
C	1.03670400	-1.79288100	0.68527400
C	-0.28177500	-2.23470300	0.85841300
C	1.94881800	-1.88394600	1.74100100
C	-0.68191500	-2.75776300	2.07868400
H	-0.97925000	-2.20309200	0.02098100

C	1.53253900	-2.38056500	2.96877600
H	2.96335000	-1.50639800	1.62510300
C	0.22224800	-2.81997800	3.13653500
H	-1.70320700	-3.11084100	2.20499300
H	2.23213200	-2.41885200	3.80076100
H	-0.09720400	-3.21360700	4.09949200
S	-3.57493900	0.50456700	-1.10800000
O	-2.45138500	-0.21898900	-1.82785700
O	-4.59573300	1.12861400	-1.90428300
C	-4.40861700	-0.82269000	-0.13702600
F	-4.95099800	-1.68317700	-0.97579700
F	-5.33646600	-0.27719600	0.62011400
F	-3.52227300	-1.44801200	0.62566400
O	-2.86349900	1.33667200	-0.06337400
S	0.90672900	2.48742400	0.82412200
O	0.90289500	2.86251500	-0.57961200
O	0.09884600	1.19160400	1.06337800
C	2.59768100	1.84880200	1.16729400
F	2.93358700	0.91820500	0.26410200
F	2.65523900	1.29667900	2.36887600
F	3.46794000	2.83686000	1.09168400
O	0.66744000	3.46199800	1.86630600
C	2.71490800	-1.61966500	-1.24519600
H	3.53528900	-1.61394900	-0.51924600
H	2.61777200	-2.65232500	-1.60760100
H	2.94689400	-0.95723900	-2.08295500

Sub4-TS1-OAc

Pd	0.63546400	0.09870800	-0.15544800
C	-1.48956600	0.38144400	0.04724300
H	-0.84213900	1.26965900	-0.73566100
O	-0.47277600	2.57456900	-1.11849100
O	1.21168200	2.02065400	0.25554600
C	0.54997400	2.85732000	-0.43660600
C	1.05641300	4.27088500	-0.43939100
C	-3.44445500	0.87858200	1.41112400
C	-4.02690800	-0.33577500	1.06493100
C	-3.33894900	-1.21516600	0.23844400
C	-2.07591100	-0.87859100	-0.25432000
O	2.56993300	-0.44120600	-0.19252900
C	2.75603400	-1.40993300	0.66182600
O	1.88406000	-1.87643300	1.38553500
C	4.18269500	-1.90818700	0.67425900
H	-3.97029700	1.56422400	2.07339700
H	-5.00760800	-0.60524100	1.45129100
H	-3.77312400	-2.18743700	0.00898400
C	-1.24838500	-1.88409200	-0.92122000
H	4.86166200	-1.08992100	0.94054000
H	4.46992900	-2.25401600	-0.32536900
H	4.28529500	-2.72425900	1.39493400
H	1.78317100	4.37400300	-1.25452300
H	1.56861900	4.49947500	0.49896900
H	0.23636400	4.97065800	-0.62122200
C	-2.19512200	1.22929400	0.90452300
H	-1.77317200	2.20289700	1.14845100
O	-0.02078900	-1.71027800	-0.99562900
C	-1.82142200	-3.14973800	-1.46869300
H	-2.11397600	-3.81301100	-0.64341700
H	-2.71475700	-2.95753900	-2.07481100
H	-1.06241600	-3.66002100	-2.06714100

Sub4-TS1-TFA

Pd	-0.55059100	0.20940700	-0.03224900
C	-0.02391000	-1.78945400	0.50485800
H	-0.90636100	-1.68821000	-0.54115100
O	-2.12456200	-1.93587500	-1.13333300
O	-2.59639500	-0.06357600	0.02363600
C	-2.90419400	-1.09473000	-0.62253600
C	-4.39762900	-1.44136500	-0.69762700
C	-0.01168000	-3.64612700	2.07572000
C	1.37656000	-3.70468100	2.03331500
C	2.08173300	-2.78993500	1.25953200
C	1.39900700	-1.82764600	0.51429000
O	-0.81956100	2.18375800	-0.37090500
C	-0.20197000	2.86827400	0.53058600
O	0.42457800	2.45909900	1.49048900
C	-0.33067000	4.38040000	0.25574200
H	-0.56683700	-4.34876300	2.69425600
H	1.91192300	-4.45106100	2.61597700
H	3.17076000	-2.80314200	1.26868100
C	2.11315900	-0.71684900	-0.11044600
C	-0.70162000	-2.69875100	1.32065300
H	-1.79126900	-2.70293100	1.33299300
O	1.47771900	0.30511500	-0.43115200
F	-5.16782100	-0.40738300	-0.41651600
F	-4.71533900	-1.90643100	-1.89619800
F	-4.61918100	-2.40281100	0.20574200
F	0.27683800	5.09401100	1.19418200
F	0.22173200	4.68420700	-0.92180300
F	-1.61248700	4.74569400	0.21636100

C	3.58734900	-0.72859300	-0.32879000
H	4.10539000	-0.63137100	0.63506900
H	3.91598400	-1.66661900	-0.79175900
H	3.86778000	0.12086100	-0.95673400

Sub4-TS1-OTf

Pd	-0.27830100	-0.02194700	0.05105900
C	0.72472000	-1.56942300	1.05884300
H	-0.18370500	-2.05907500	0.15053600
C	1.27255500	-2.74904600	3.10802700
C	2.63090100	-2.59021300	2.85841700
C	3.05615300	-1.88688900	1.73410100
C	2.11853500	-1.36498600	0.84647200
H	0.94219100	-3.28008000	3.99847600
H	3.36396800	-2.99744800	3.55127400
H	4.11972600	-1.71584400	1.57355400
C	2.49741000	-0.43105800	-0.21139500
C	0.32678700	-2.23518000	2.22050800
H	-0.73338400	-2.39138800	2.41710500
O	1.60957900	0.27057200	-0.73332100
S	-2.51955700	-2.03075300	-0.01889700
O	-1.22449000	-2.74928400	-0.34365900
O	-2.20206200	-0.68538900	0.58746700
C	-3.19491800	-1.62459200	-1.68392300
F	-2.31007800	-0.90288200	-2.34616400
F	-4.31032800	-0.94393100	-1.53015600
F	-3.43298500	-2.74892300	-2.33105800
O	-3.51487600	-2.80156100	0.68513400
S	-0.57008600	2.95139600	-0.10732500
O	-1.02580100	1.67100500	-0.82615700
O	-0.53497800	4.08242100	-1.01025200
C	-1.99894800	3.23758400	1.01053200
F	-3.10782400	3.39015600	0.30860800
F	-1.78020900	4.32626100	1.73097500
F	-2.13869400	2.19872500	1.82729100
O	0.55457300	2.68142200	0.78524900
C	3.90481300	-0.23767100	-0.65485100
H	4.47117300	0.27702600	0.13344300
H	4.39709600	-1.19825400	-0.84842800
H	3.92211600	0.38458200	-1.55314800

Sub5

C	2.46283800	1.02162900	-0.00003400
C	1.08843400	1.21513900	-0.00002600
C	0.22729800	0.11609100	-0.00001000
C	0.74923000	-1.17917600	-0.00000200
C	2.12542800	-1.36923400	-0.00001100
C	2.98160600	-0.27101600	-0.00002700
H	3.13309200	1.87933600	-0.00004600
H	0.65442600	2.21303700	-0.00003100
H	0.07215800	-2.03000200	0.00001000
H	2.53308600	-2.37862200	-0.00000600
H	4.05986400	-0.42359100	-0.00003300
C	-1.23057500	0.38671000	-0.00000100
O	-1.72463700	1.49203200	0.00000100
O	-1.96146300	-0.74305700	0.00003300
C	-3.36890800	-0.54374000	0.00005400
H	-3.68079300	0.01514500	-0.88920200
H	-3.81438500	-1.54076300	0.00011400
H	-3.68075400	0.01523700	0.88926400

Sub5-INT1-OAc

Pd	-1.17030200	-0.47462200	0.22927300
C	0.82810200	2.17966800	0.35186200
O	-2.31339200	1.16295200	0.90362000
O	-2.98936500	-0.16369600	-0.68901200
C	-3.20864800	0.84603300	0.05787500
C	-4.48603900	1.60203200	-0.04327500
C	2.10619200	3.50859400	-1.19911600
C	3.05576100	2.49392300	-1.29923700
C	2.90137900	1.32466000	-0.56912000
C	1.78340500	1.16527400	0.25279400
O	-0.62778500	-2.14222100	-0.71489800
C	0.57528100	-2.31046700	-1.17762200
O	1.54319400	-1.57603000	-1.00386400
C	0.67475500	-3.57027200	-2.01250100
H	2.23182900	4.42675000	-1.77088800
H	3.91687700	2.61450000	-1.95361200
H	3.61930200	0.51241900	-0.65120100
C	1.61572700	-0.06793600	1.03804400
H	0.09333600	-3.44160500	-2.93303600
H	0.24470300	-4.42296800	-1.47611300
H	1.71993500	-3.76731100	-2.26658500
H	-5.23630800	1.12651400	0.60056200
H	-4.85884000	1.57988700	-1.07176700
H	-4.34440900	2.63223300	0.29653900
C	0.99692900	3.35315500	-0.37273200
H	0.25938500	4.14900400	-0.28996700
O	0.53570400	-0.53466500	1.43366500
O	2.75153100	-0.60672800	1.42355300
C	2.66560600	-1.88485400	2.05865600

H	3.69739000	-2.19084800	2.23774900
H	2.11583600	-1.80845500	3.00141700
H	2.16081400	-2.58815200	1.38903400
H	-0.03574900	2.05488300	1.00304700

Sub5-INT1-TFA

Pd	0.48394000	-0.50286400	-0.58231900
C	0.62009300	2.76279200	0.11937000
O	2.45542100	0.24858300	-0.82607300
O	2.03722900	-1.64621200	0.19296000
C	2.85867800	-0.77957200	-0.21695800
C	4.35885800	-0.95567100	0.01376100
C	0.42650800	4.08596500	2.12154900
C	-0.94852500	3.86152300	2.14963700
C	-1.54714200	3.09587200	1.16007700
C	-0.75962700	2.53997100	0.14846200
O	-1.06803700	-1.69815300	-0.21010500
C	-2.11279700	-1.21022500	0.35792100
O	-2.33679400	-0.07769900	0.74801600
C	-3.24900900	-2.25371600	0.43428400
H	0.89193200	4.68970300	2.89878400
H	-1.55408000	4.28134800	2.95009100
H	-2.61469400	2.89014300	1.17767400
C	-1.38109900	1.72177800	-0.89929600
C	1.20891500	3.54223800	1.10634000
H	2.28092900	3.72573500	1.08105900
O	-0.82338300	0.79727600	-1.52256900
O	-2.59446600	2.09366300	-1.22323000
C	-3.32518800	1.24908000	-2.12385300
H	-4.28142900	1.75081500	-2.27535200
H	-2.78759900	1.14268100	-3.06980100
H	-3.47492900	0.26786600	-1.66226900
H	1.22498800	2.34456000	-0.68302800
F	-4.05096900	-2.00483500	1.45987800
F	-3.97132100	-2.14728500	-0.69343300
F	-2.81407500	-3.50167300	0.52948300
F	4.98187900	-0.99910100	-1.15950900
F	4.61510200	-2.06503200	0.68383100
F	4.82197000	0.08584800	0.69947000

Sub5-INT1-OTf

C	1.17816900	-1.23350300	-0.93595600
O	0.44558400	-0.38224300	-1.46205500
Pd	-1.10024000	0.53715500	-0.46197800
C	0.84016100	-1.89062700	0.34399300
C	-0.43768900	-2.44411400	0.47514600
C	1.71872800	-1.87575200	1.42942400
C	-0.82431200	-3.00173700	1.68647100
H	-1.11811700	-2.44586800	-0.37701500
C	1.31413000	-2.41392800	2.64359700
H	2.68890700	-1.39030800	1.34222600
C	0.04919000	-2.98149300	2.77030200
H	-1.81554000	-3.43858500	1.78670200
H	1.98520900	-2.37768200	3.49882000
H	-0.26186300	-3.40242900	3.72436100
S	-3.73210300	0.71936100	-0.83933500
O	-2.71234100	0.02650100	-1.72363100
O	-4.79052400	1.45687700	-1.47262000
C	-4.53493900	-0.66215600	0.07976400
F	-5.19989500	-1.41005600	-0.77894200
F	-5.35646800	-0.15722800	0.97561600
F	-3.61238500	-1.39675900	0.68380200
O	-2.88968800	1.42041100	0.20614900
S	0.97631600	2.38395600	0.79011200
O	0.85401500	2.85566100	-0.57801100
O	0.14802200	1.09825700	1.01950800
C	2.66899400	1.67554800	0.92884500
F	2.86373500	0.73706100	-0.00562800
F	2.84166400	1.11332700	2.11722100
F	3.56325300	2.62948500	0.75914500
O	0.87115000	3.29024900	1.91324800
O	2.25571600	-1.54045800	-1.61572700
C	3.14792300	-2.58578200	-1.22616700
H	2.62118900	-3.39316700	-0.70828100
H	3.59019500	-2.95529600	-2.15329400
H	3.93271900	-2.17142800	-0.58484400

Sub5-TS1-OAc

Pd	0.71633500	-0.00625300	-0.07266500
C	-1.16581200	1.04223200	0.10114400
H	-0.28072300	1.55792600	-0.77641300
O	0.51373400	2.58987000	-1.29892000
O	1.93541700	1.62323900	0.14218100
C	1.58943800	2.56335500	-0.64108900
C	2.55802200	3.70132600	-0.78761400
C	-2.76004900	2.33627700	1.40900000
C	-3.74485500	1.37517100	1.20632900
C	-3.44710500	0.22597400	0.48412600
C	-2.17187800	0.05606200	-0.05188400
O	2.34599200	-1.17956800	-0.07099400
C	2.21487200	-2.09432000	0.85024100

O	1.25038800	-2.19462000	1.59959100
C	3.39858100	-3.03202800	0.91086000
H	-2.98321300	3.22806500	1.99228400
H	-4.73912000	1.51072600	1.62693100
H	-4.18799800	-0.56118400	0.36098500
C	-1.76890600	-1.23404500	-0.61801000
H	4.29843200	-2.47326300	1.19372800
H	3.58812400	-3.47142600	-0.07502900
H	3.21116100	-3.82104400	1.64460700
H	3.23971800	3.46844500	-1.61480400
H	3.15606100	3.81934200	0.11979300
H	2.02703300	4.62517300	-1.03235100
C	-1.49008800	2.16875100	0.86300500
H	-0.74308200	2.94947200	0.99642700
O	-0.58021300	-1.55073800	-0.75041900
O	-2.73946800	-2.06540100	-0.93735300
C	-2.33416600	-3.36425900	-1.38859700
H	-3.25956200	-3.91721700	-1.55398800
H	-1.76244700	-3.27835700	-2.31765400
H	-1.71647800	-3.85030000	-0.62734200

Sub5-TS1-TFA

Pd	0.72955800	-0.04569100	-0.06738700
C	-1.11493900	1.04113300	0.03270800
H	-0.21393900	1.53407800	-0.87541400
O	0.60195000	2.52211400	-1.36571800
O	1.95720000	1.59262200	0.17266600
C	1.61156200	2.50266500	-0.61998500
C	2.44347900	3.79250100	-0.59293000
C	-2.71087200	2.43517400	1.22610600
C	-3.71542000	1.49392200	1.03028300
C	-3.42912300	0.30293900	0.37182300
C	-2.14349700	0.07528700	-0.11114900
O	2.34413300	-1.26111600	-0.02930200
C	2.19435200	-2.16984200	0.87278600
O	1.27304800	-2.31414500	1.65492800
C	3.38165900	-3.15440400	0.86065200
H	-2.92770200	3.35858200	1.75979500
H	-4.71877100	1.67738000	1.40846600
H	-4.18815700	-0.46850000	0.26008600
C	-1.74725600	-1.24624400	-0.60052300
C	-1.42580100	2.20874100	0.73702000
H	-0.66665400	2.97987600	0.86476700
O	-0.55286200	-1.57517300	-0.68760400
O	-2.70806500	-2.08717800	-0.89691000
C	-2.30287700	-3.41468600	-1.27217800
H	-3.23002500	-3.96640200	-1.42843000
H	-1.71105900	-3.37826100	-2.19139400
H	-1.70686800	-3.86253600	-0.47165000
F	3.26233800	-4.06534600	1.81744000
F	4.53565800	-2.51111700	1.03671600
F	3.43821600	-3.79013600	-0.31375700
F	3.62582500	3.61530100	-0.03424300
F	2.60868100	4.27324700	-1.81577100
F	1.75503800	4.68466800	0.12732100

Sub5-TS1-OTf

Pd	-0.22766600	-0.11668300	-0.13695100
C	-2.16930200	0.24140400	0.60014300
C	-3.72428700	0.45958000	2.45131100
C	-4.55082800	-0.51085000	1.89532400
C	-4.18203500	-1.15228600	0.71514900
C	-3.00178200	-0.78526300	0.08088900
H	-4.00348800	0.93987700	3.38696200
H	-5.47654200	-0.78870600	2.39466800
H	-4.78761800	-1.95727100	0.30403900
C	-2.44695200	-1.56514800	-1.02719000
C	-2.53678000	0.82337000	1.81811100
H	-1.91314100	1.60141300	2.25687000
O	-1.26096100	-1.42922100	-1.37586600
O	-3.22798400	-2.44202200	-1.60093200
C	-2.61765800	-3.28852800	-2.59515200
H	-1.75911700	-3.80867900	-2.16024000
H	-3.39727000	-3.98935300	-2.89332100
H	-2.28865400	-2.68396900	-3.44507800
H	-1.67935200	1.33856700	-0.06644800
S	0.06769300	2.83683900	0.35611100
O	0.61913800	1.52494200	0.86103600
O	0.12864800	3.92823800	1.29731600
C	1.15180300	3.26228000	-1.07132500
F	2.37623300	3.44816800	-0.62691200
F	0.69864600	4.36623600	-1.63291900
F	1.13031800	2.27192100	-1.94301500
O	-1.27703400	2.59351400	-0.30070600
S	1.98481700	-2.13533500	-0.30136600
O	2.88975300	-2.80740800	-1.21050400
O	0.82355200	-2.84103700	0.23577900
C	2.98345600	-1.68368200	1.17227200
F	3.40177200	-2.78607800	1.77369300
F	2.23372800	-0.98580000	2.01897500

F	4.02589300	-0.95536800	0.81404700
O	1.60131200	-0.74115000	-0.82020700

Tri-Pd-OAc

Pd	1.77947300	0.34790700	0.00490800
C	1.64302300	-1.96340100	1.89521500
O	2.37417000	-1.17295900	1.23884100
O	0.42111500	-2.21696900	1.70559900
C	-2.51599300	-0.57069700	-1.87245000
O	-2.59347500	0.51876200	-1.24392700
O	-1.70229500	-1.51667000	-1.67564800
C	-3.53048100	-0.78606400	-2.96219700
H	-3.14442900	-1.48458400	-3.70948200
H	-4.43176300	-1.22531900	-2.51701800
H	-3.80412400	0.16696900	-3.42272800
C	2.31178000	-2.67634700	3.03861000
H	2.58679500	-1.94152900	3.80386000
H	1.65105500	-3.43204000	3.46963800
H	3.23865300	-3.14021500	2.68479900
Pd	-1.18653500	1.36771400	-0.02229200
C	-2.55565600	-0.41953800	1.85366900
O	-2.21639600	-1.45794200	1.22550900
O	-2.15187900	0.76205600	1.66241900
C	1.75082900	-1.92392800	-1.84153500
O	2.16156100	-0.74210800	-1.66877300
O	0.85096700	-2.52630300	-1.19682800
C	-3.58596600	-0.59966700	2.93491700
H	-3.50134500	-1.59341100	3.38252100
H	-3.48173200	0.18088400	3.69353300
H	-4.58215900	-0.50647300	2.48509800
Pd	-0.58686300	-1.71287600	0.01272700
C	0.79478300	2.44259900	-1.88922600
O	1.76471300	1.98190200	-1.22836500
O	-0.43443400	2.21895100	-1.70842100
C	1.15502800	3.35931100	-3.02624100
H	1.70422900	2.78851300	-3.78363100
H	0.26116600	3.80342600	-3.47030800
H	1.82586700	4.14391600	-2.66020200
C	0.88327000	2.43573700	1.85156700
O	1.71190800	1.49879000	1.68074400
O	-0.16632900	2.66282900	1.19080700
C	1.17148300	3.39773400	2.97166400
H	1.18035200	4.41918200	2.57581300
H	0.36322500	3.34168900	3.70931500
H	2.12911000	3.17143700	3.44617600
C	2.43404700	-2.71686300	-2.92213300
H	2.95563700	-2.05308100	-3.61630500
H	3.16995800	-3.38186900	-2.45376900
H	1.70620400	-3.33978000	-3.44992900

Tri-Pd-TFA

Pd	0.00023800	-1.67794800	0.79373100
C	-1.83986200	-2.10004000	-1.51610600
O	-1.27505600	-2.58921000	-0.51231200
O	-1.63377300	-1.02569300	-2.12208500
C	1.83937300	2.31876900	-1.15399200
O	1.27439000	2.63799000	-0.08406100
O	1.63313600	1.35761600	-1.92704400
C	2.97177300	3.28926600	-1.55953400
C	-2.97131700	-2.99219700	-2.07513700
Pd	-0.00086700	1.52612300	1.05625800
C	-1.84093100	2.36267200	-1.06015600
O	-1.27605900	1.73804700	-1.98571500
O	-1.63466900	2.35045400	0.17324000
C	1.84050600	-2.15840500	-1.43052600
O	1.63401400	-2.34752100	-0.21169500
O	1.27554700	-1.39143800	-2.24198600
C	2.97344000	-2.99435200	-2.06799000
C	-2.97293500	3.29224800	-1.55323700
Pd	-0.00030300	0.15165600	-1.84955500
C	1.84006700	-0.15904600	2.58476400
O	1.27572100	-1.24560600	2.32630800
O	1.63292200	0.99093800	2.13915600
C	2.97307300	-0.29257700	3.62742500
C	-1.84027400	-0.26406100	2.57663700
O	-1.63345600	-1.32607100	1.94953600
O	-1.27608900	0.85014700	2.49822500
C	-2.97258200	-0.30217900	3.62787700
F	2.46981900	-0.79769400	4.74712300
F	3.89717100	-1.12163200	3.15810900
F	3.53219700	0.87334800	3.89178100
F	3.53314600	-3.80606000	-1.19061600
F	2.46991400	-3.71169200	-3.06500100
F	3.89712600	-2.17323700	-2.55178900
F	3.89514800	3.29918900	-0.60626200
F	3.53216100	2.93485200	-2.70072200
F	2.46726900	4.51087100	-1.68340800
F	-2.46487000	-4.17569500	-2.39936300
F	-3.89363300	-3.16109300	-1.13588300
F	-3.53351200	-2.45529800	-3.14173700

F	-2.46855300	0.01421300	4.81449800
F	-3.53135300	-1.49570700	3.69947200
F	-3.89713200	0.59193600	3.30034300
F	-3.89560600	2.56239700	-2.16753500
F	-3.53436700	3.94847000	-0.55520100
F	-2.46738100	4.16394200	-2.41744100

Tri-Pd-OTf

Pd	1.15126000	1.27017200	-0.44421400
Pd	0.56847500	-1.59931800	0.50363700
Pd	-2.37138000	0.41225200	-0.18648600
C	-1.58760600	4.34391700	1.61161300
S	-0.98486900	2.60164000	1.49800400
O	-0.16072200	2.69957400	0.22970300
O	-2.28036400	1.85631800	1.24621000
O	-0.29543900	2.23833400	2.70811000
F	-0.54427200	5.13113300	1.76461900
F	-2.23438600	4.64908500	0.50766700
F	-2.38583200	4.43242600	2.65240000
C	-2.25273000	-2.85015000	2.97065700
S	-1.94453800	-1.09159500	2.49233300
O	-2.78455200	-0.99798000	1.23791600
O	-0.46278800	-1.03090800	2.18178600
O	-2.31920400	-0.26431400	3.60785700
F	-3.54048400	-3.00025200	3.19381900
F	-1.86074100	-3.65821600	2.00633200
F	-1.56560300	-3.09115500	4.06716200
C	4.45260600	0.31268900	2.01307800
S	2.60562600	0.30535600	2.14195300
O	2.29172000	-1.01188500	1.47322000
O	2.22524100	1.48682400	1.26730300
O	2.25868000	0.44088700	3.53128800
F	4.91643100	-0.81078300	2.51882300
F	4.81419500	0.42576800	0.75181500
F	4.89701800	1.34266700	2.70176300
C	-2.90704000	-3.56326500	-1.74935000
S	-1.65359100	-2.20761100	-1.72448400
O	-1.02629500	-2.51351200	-0.38376700
O	-2.54036400	-0.97927300	-1.65632600
O	-0.82220800	-2.27808300	-2.89681300
F	-2.27556700	-4.71752300	-1.77942000
F	-3.64841700	-3.47494800	-0.66493300
F	-3.64902300	-3.41860900	-2.82557200
C	-0.47676600	3.46975000	-2.88302200
S	-1.08423700	1.69447500	-2.69758600
O	-2.14660500	1.84035600	-1.63243700
O	0.11200300	0.92291000	-2.17396500
O	-1.52053400	1.23123100	-3.98753700
F	-0.65200800	3.82571100	-4.13358400
F	-1.14408700	4.26799100	-2.08360900
F	0.81059300	3.50889500	-2.58111400
C	4.22713600	-1.91464600	-1.57806000
S	2.54965600	-1.20714300	-1.90827500
O	2.67782900	0.13491000	-1.22652000
O	1.65206800	-2.14673800	-1.12627300
O	2.33394000	-1.20942500	-3.33019000
F	5.13807300	-1.06133100	-1.99575400
F	4.36759700	-2.13534500	-0.28735900
F	4.32252600	-3.04756200	-2.24108700

OAc

O	-0.74818600	-1.13336700	0.00281200
O	-0.74546700	1.13473300	0.00284000
C	-0.21941700	0.00007600	-0.01277600
C	1.34367300	-0.00118600	-0.00499400
H	1.70222700	-0.00158700	1.03714300
H	1.75103600	0.89917600	-0.48785700
H	1.75041900	-0.90185700	-0.48787900

TFA

O	1.50914800	-1.14468700	-0.00004700
O	1.58357200	1.13477700	-0.00005600
C	1.05559000	0.01228600	-0.00010900
C	-0.51105900	0.01626100	-0.00003000
F	-1.07790700	1.23799000	-0.00113800
F	-1.01724900	-0.62514600	-1.07781000
F	-1.01694800	-0.62306600	1.07913100

OTf

S	-0.91135500	0.00014500	-0.00014900
O	-1.22130600	-1.10788100	-0.91409600
O	-1.22165000	-0.23748000	1.41635900
C	0.92711100	0.00003600	0.00002300
F	1.41890000	-1.16460000	0.43832900
F	1.41992100	0.96149800	0.78947400
F	1.42043400	0.20254500	-1.22721600
O	-1.22132800	1.34566900	-0.50264300

Sub5-TS2-PdOAc-OAc

Pd	-0.70393000	-0.17332800	0.39416800
C	0.74272900	1.27685100	-0.25590500
H	1.00410300	0.92866000	0.92494100
O	-3.01497600	1.29709800	-0.85786300

O	-1.99343300	1.16723100	1.15283500
C	-2.91920300	1.56318300	0.33016700
C	-3.94248000	2.44348800	1.03469800
C	0.92941400	3.28127600	-1.59959300
C	1.71127800	2.59942100	-2.53149500
C	2.00878300	1.25162100	-2.34924000
C	1.51827400	0.60347900	-1.22368900
O	-2.13978500	-1.58979700	0.66014700
C	-2.39903800	-2.26982900	-0.41388600
O	-1.82904200	-2.19438000	-1.49424000
C	-3.56591800	-3.22595600	-0.20462800
H	0.69245400	4.33266100	-1.76188200
H	2.08410000	3.12090100	-3.41260000
H	2.59933200	0.70211100	-3.08030800
C	1.67828400	-0.83673600	-0.97592400
H	-4.49506400	-2.64732500	-0.12783800
H	-3.45061900	-3.78337500	0.73231700
H	-3.64206400	-3.91719000	-1.05032400
H	-3.44688000	3.18967100	1.66670300
H	-4.56160900	1.82150800	1.69317900
H	-4.58376700	2.93609000	0.29680500
C	0.44763300	2.62540700	-0.47134500
H	-0.16296200	3.15191300	0.26111600
O	0.87685500	-1.46480300	-0.27613500
O	2.66109300	-1.43336600	-1.62502800
C	2.89623900	-2.78466900	-1.24016900
H	3.73404100	-3.12643000	-1.85260200
H	3.15644000	-2.80565900	-0.17643600
H	2.00726200	-3.39832000	-1.42024400
O	3.26651700	-0.43017500	0.97682900
C	2.83192300	0.07943200	2.02170400
O	1.76286400	0.76420000	2.16548700
C	3.61797900	-0.10777300	3.31594900
H	4.57364500	-0.60814400	3.12525000
H	3.02511700	-0.70981700	4.01661800
H	3.79109200	0.86261200	3.79744600

Sub5-TS2-PdOAc-TFA

Pd	1.04404700	0.21790600	0.10025700
C	-0.77293700	1.31546700	0.03434500
H	-0.08299500	1.54444300	-1.05995400
O	1.50369400	0.87127700	2.99013300
O	2.10649900	1.68366600	0.97095800
C	2.13553100	1.62900900	2.27060900
C	3.09521500	2.66112800	2.84478100
C	-2.32075500	2.62888300	1.35233200
C	-3.32444200	1.68303300	1.14398800
C	-3.06867000	0.54184200	0.38997300
C	-1.80017200	0.36480800	-0.14856900
O	2.61872200	-1.02500600	0.44029400
C	2.28178900	-2.11734900	1.05398000
O	1.15015500	-2.47583100	1.35406200
C	3.49627500	-2.96437100	1.40647200
H	-2.53169100	3.51359500	1.95282900
H	-4.31216500	1.83464600	1.57790600
H	-3.83740800	-0.21287600	0.23303300
C	-1.38785300	-0.83088700	-0.89510200
H	4.05975000	-2.46895200	2.20710500
H	4.16839600	-3.05909300	0.54560900
H	3.17822400	-3.95403100	1.75007200
H	2.95534700	3.63580900	2.36299800
H	4.12662600	2.34582500	2.64383300
H	2.94886000	2.74703700	3.92616100
C	-1.05539500	2.44709900	0.80366400
H	-0.26891400	3.18414700	0.96114200
O	-0.19876800	-1.14006100	-1.01754700
O	-2.35984600	-1.57838200	-1.38376600
C	-1.93096200	-2.63380900	-2.24176100
H	-2.84181700	-3.14694300	-2.55804300
H	-1.40898700	-2.20111300	-3.10205000
H	-1.26330200	-3.31737000	-1.70723100
O	-1.30103300	0.51439000	-3.17043500
C	-0.46973700	1.41671200	-3.24580100
O	0.19548200	2.01004800	-2.34308300
C	-0.19748200	1.93442500	-4.67777700
F	0.76073800	2.85725900	-4.75422900
F	0.15454800	0.92302300	-5.48291300
F	-1.31438700	2.47536200	-5.19052500

Sub5-TS2-PdOAc-OTf

Pd	0.88719800	0.36887200	-0.01782900
C	-0.96570800	1.31066600	0.20756400
H	-0.33421100	1.76273100	-0.95727000
O	1.61078200	0.79928900	2.85036800
O	1.91995000	1.84536300	0.87354000
C	2.09899500	1.67324500	2.15126500
C	3.03441000	2.72714500	2.72455000
C	-2.51702200	2.48417300	1.64727500
C	-3.50796900	1.53890800	1.37968800
C	-3.24384700	0.46879200	0.53235600

C	-1.97919400	0.35862300	-0.03604000
O	2.57998400	-0.76847200	0.03865800
C	2.38108200	-1.93772300	0.56222600
O	1.30923800	-2.40789300	0.92529800
C	3.67589300	-2.72242300	0.71429000
H	-2.73451800	3.31581400	2.31731200
H	-4.49157200	1.63816800	1.83706100
H	-4.00271300	-0.28283700	0.32172000
C	-1.56480100	-0.75217400	-0.89780600
H	4.27814200	-2.26981000	1.51210200
H	4.26898200	-2.67759800	-0.20661500
H	3.45839300	-3.76322000	0.97534800
H	2.76740200	3.72618200	2.36086900
H	4.05790500	2.52258400	2.38671000
H	3.00361700	2.69971800	3.81832700
C	-1.25752000	2.37233800	1.06760900
H	-0.48360100	3.11302900	1.26615200
O	-0.37537200	-0.97702000	-1.13983400
O	-2.52845800	-1.50881100	-1.39261200
C	-2.09464800	-2.48859800	-2.33449100
H	-2.99964500	-3.00302100	-2.66441000
H	-1.60212100	-1.98930500	-3.17639900
H	-1.39487100	-3.18800600	-1.86547500
C	0.55148800	1.17391000	-4.17054200
S	-0.85626000	1.97647200	-3.31040200
O	-1.24892200	3.06858200	-4.18481500
O	-0.18751000	2.46830100	-2.03613400
O	-1.80468700	0.88878300	-3.07622000
F	0.13147300	0.69494800	-5.34077400
F	1.52121700	2.04939200	-4.39551400
F	1.02998200	0.17057600	-3.45200000

Sub5-TS2-PdTFA-OAc

Pd	0.00950900	0.09515300	0.33166400
C	-1.49337400	-1.29391800	-0.33838200
H	-1.68958300	-1.04416200	0.83184200
O	2.25417700	-1.28336300	-1.21926200
O	1.30877600	-1.35595200	0.84506000
C	2.11419700	-1.71124800	-0.09160800
C	2.93380600	-2.93558200	0.37172500
C	-1.66644100	-3.14514500	-1.88155900
C	-2.46186100	-2.37341700	-2.72755000
C	-2.76468500	-1.05086500	-2.40949000
C	-2.27442800	-0.52048800	-1.22575100
O	1.46580500	1.49748200	0.64907800
C	1.67571800	2.24826500	-0.36926200
O	1.13704200	2.27359700	-1.45989200
C	2.79647900	3.26503600	-0.05208300
H	-1.42109200	-4.16970400	-2.15777100
H	-2.83703300	-2.80220800	-3.65602800
H	-3.35544600	-0.43158500	-3.08172100
C	-2.40154300	0.89696500	-0.85019100
C	-1.17938500	-2.60881400	-0.69408600
H	-0.54673400	-3.19902600	-0.03166800
O	-1.54216100	1.44928800	-0.14519000
O	-3.37077300	1.57144500	-1.42710200
C	-3.55538600	2.89967900	-0.93820500
H	-4.39936100	3.30621800	-1.49938200
H	-3.78510800	2.84618300	0.13094200
H	-2.65371200	3.49864600	-1.10200700
O	-3.91397200	0.39622200	1.02839800
C	-3.48767100	-0.15751700	2.06056500
O	-2.43429100	-0.86066700	2.17759200
C	-4.27986800	0.00713600	3.35350600
F	3.08075300	4.03605800	-1.10121900
F	2.43313300	4.07568300	0.95244000
F	3.92702100	2.65403200	0.31653700
F	3.88419300	-3.25900600	-0.50156400
F	3.51775900	-2.74485600	1.55596200
F	2.11924600	-3.99988400	0.49130100
H	-5.22976600	0.52123500	3.17110500
H	-3.68496300	0.58691400	4.07099200
H	-4.46374600	-0.97272000	3.81082300

Sub5-TS2-PdTFA-TFA

Pd	1.08165200	0.19469400	0.09480700
C	-0.69695400	1.34098800	0.02784900
H	-0.03517200	1.54999700	-1.04970700
O	1.54073300	0.80095500	3.03894800
O	2.12258400	1.65326300	1.01603500
C	2.07715900	1.60139500	2.30026900
C	2.78027500	2.84235500	2.89199300
C	-2.18561300	2.66731200	1.39278000
C	-3.21087200	1.74428700	1.18901500
C	-2.99430700	0.60186100	0.42230900
C	-1.74203500	0.40560500	-0.14202900
O	2.63512800	-1.10176000	0.41400100
C	2.27549800	-2.17365800	1.01883200
O	1.17589600	-2.53859700	1.39248500
C	3.49787100	-3.09227700	1.24440700

H	-2.36728200	3.54667000	2.00929300
H	-4.18696100	1.91145600	1.64239000
H	-3.78012800	-0.13736100	0.27854600
C	-1.35429400	-0.80054500	-0.88607900
C	-0.93295300	2.46732000	0.82097000
H	-0.12362400	3.17885300	0.98371500
O	-0.16501700	-1.13059300	-0.99794200
O	-2.33133700	-1.54062600	-1.35846700
C	-1.92380900	-2.61415600	-2.20951300
H	-2.84529600	-3.11390100	-2.51414800
H	-1.40098200	-2.19570600	-3.07583700
H	-1.26549100	-3.30211400	-1.66973800
O	-1.28703600	0.49423000	-3.12814900
C	-0.43238500	1.37174200	-3.25381100
O	0.26811400	1.97082200	-2.38484600
C	-0.18364000	1.83369400	-4.70855300
F	0.79643900	2.72589400	-4.83673200
F	0.12043600	0.78599700	-5.48554000
F	-1.29874400	2.38729300	-5.20992700
F	3.17230800	-4.19925200	1.91091900
F	4.02921600	-3.47213800	0.07350600
F	4.45829800	-2.47219600	1.93721900
F	2.88554800	2.77032200	4.21603900
F	4.00439900	3.02399700	2.39496500
F	2.06090600	3.94246100	2.60478400

Sub5-TS2-PdTFA-OTf

Pd	0.93651100	0.35612900	-0.02624600
C	-0.88721400	1.35319800	0.18793800
H	-0.28733000	1.76327500	-0.95668500
O	1.64866800	0.73885900	2.89296500
O	1.96160100	1.81255600	0.91722700
C	2.05486600	1.64057800	2.18879400
C	2.73915000	2.86829600	2.82777300
C	-2.38572700	2.55409800	1.65370000
C	-3.39536500	1.62500100	1.40336400
C	-3.16636100	0.54323700	0.55811400
C	-1.91595600	0.40993600	-0.03117000
O	2.60332700	-0.84039800	0.02322300
C	2.37697100	-1.98703400	0.54909600
O	1.34359300	-2.46660500	0.97988800
C	3.67341300	-2.82750000	0.57789000
H	-2.57741000	3.38909400	2.32635200
H	-4.36889800	1.74278200	1.87693100
H	-3.94068700	-0.19774300	0.36829100
C	-1.52196800	-0.71798700	-0.88089500
C	-1.13701900	2.42059200	1.05422600
H	-0.34440600	3.14381200	1.24569100
O	-0.32975800	-0.96317900	-1.11017600
O	-2.48592600	-1.46925400	-1.36541700
C	-2.06226300	-2.45921700	-2.30667400
H	-2.97580400	-2.94615000	-2.65222400
H	-1.54249500	-1.96759500	-3.13651700
H	-1.39055700	-3.17803900	-1.82674800
C	0.50484300	1.03665300	-4.22101600
S	-0.83548600	1.92191500	-3.33489900
O	-1.23024800	2.99388900	-4.23193400
O	-0.10249700	2.42470400	-2.10415800
O	-1.81066100	0.87450400	-3.02534800
F	0.01820200	0.52554700	-5.35006600
F	1.49419600	1.86496900	-4.51980700
F	0.97747900	0.04422200	-3.48019000
F	3.48675700	-4.01229000	1.15862200
F	4.11786700	-3.05744600	-0.66605800
F	4.65024000	-2.20211300	1.24252600
F	2.98574500	2.68238700	4.12137700
F	3.89136400	3.17923900	2.23308100
F	1.92855300	3.93658700	2.72156900

Sub5-TS2-PdOTf-OAc

Pd	0.13119300	0.49815400	0.12259200
C	-1.57335800	1.70540600	0.63022500
C	-2.66030400	1.99287200	2.77253000
C	-3.65417700	1.09149000	2.39003100
C	-3.62116900	0.46649300	1.14365100
C	-2.58531400	0.77275700	0.27769000
H	-2.68652000	2.44180100	3.76355200
H	-4.45696700	0.85140300	3.08595000
H	-4.37044500	-0.27016000	0.86165800
C	-2.32810600	0.05449300	-0.98093700
C	-1.62376700	2.29898700	1.90074800
H	-0.83365700	2.98485600	2.20056100
O	-1.15161500	-0.08741600	-1.37588500
O	-3.32346200	-0.59469400	-1.52231100
C	-2.99684900	-1.30333800	-2.72373200
H	-2.20016900	-2.02766100	-2.52447300
H	-3.91881900	-1.80126800	-3.02906900
H	-2.67409900	-0.57952000	-3.47893900
H	-1.13881900	2.31290600	-0.28383700
S	1.14907200	0.69109200	3.00559400

O	1.32518800	1.26202400	1.59921600
O	0.30402900	-0.49375600	3.03943000
C	2.85998900	0.10397800	3.31990900
F	3.23212500	-0.77028100	2.40301800
F	2.90914100	-0.47448400	4.51660000
F	3.70408100	1.12844000	3.30920100
O	0.92880300	1.74461600	3.98916800
S	1.13692100	-2.37166100	-0.63392200
O	1.73556900	-2.88714100	-1.85747500
O	-0.28292300	-2.61579500	-0.39429100
C	1.98667100	-3.28292500	0.71423300
F	1.78680900	-4.59007900	0.54716300
F	1.50777500	-2.93091000	1.89458100
F	3.29364700	-3.05167000	0.68695300
O	1.58119800	-0.94816000	-0.33287600
C	-2.00509500	3.75581700	-3.68616000
C	-1.94995600	2.87830100	-2.44039900
O	-1.02147900	3.16811800	-1.62680400
O	-2.79527100	1.96363000	-2.32769900
H	-1.10656600	3.58039300	-4.29166100
H	-1.99630100	4.81497000	-3.40158900
H	-2.89427500	3.53428400	-4.28602100

Sub5-TS2-PdOTf -TFA

Pd	0.07457400	0.38036500	0.11902800
C	-1.62321900	1.49964600	0.69981000
C	-2.86765000	2.05853000	2.69374100
C	-3.92862900	1.26369600	2.26202200
C	-3.85310100	0.55834100	1.06146000
C	-2.70446500	0.67498900	0.29717100
H	-2.93703700	2.58318900	3.64481300
H	-4.82316400	1.17944000	2.87736000
H	-4.66457900	-0.08855600	0.73433200
C	-2.44057400	-0.09751500	-0.92281700
C	-1.71472800	2.17345500	1.92405400
H	-0.87801100	2.78107000	2.26506200
O	-1.27230500	-0.27118100	-1.31415600
O	-3.45773700	-0.66665500	-1.51171400
C	-3.13984200	-1.38676100	-2.71097400
H	-2.39330100	-2.15829700	-2.49650300
H	-4.08105800	-1.82577600	-3.04528200
H	-2.75100600	-0.68098200	-3.45182200
H	-1.12393200	2.13466400	-0.24341200
S	1.26845400	0.71695600	2.92643300
O	1.30886800	1.25276400	1.49465300

O	0.48184000	-0.50066900	3.05604300
C	3.02265000	0.21401300	3.12784900
F	3.36047000	-0.68267600	2.21979200
F	3.18658300	-0.31080200	4.33866000
F	3.81679700	1.27050700	3.01132200
O	1.07459700	1.79304100	3.89027800
S	1.13563700	-2.46675200	-0.59933300
O	1.67323800	-2.99858000	-1.84346700
O	-0.25954500	-2.74028600	-0.26590100
C	2.09827700	-3.30836400	0.71776500
F	1.93186700	-4.62527200	0.60139500
F	1.68358800	-2.93436400	1.91523900
F	3.39170800	-3.03544200	0.59910100
O	1.54916800	-1.02067600	-0.36487400
C	-1.72223700	3.88871200	-3.43259800
C	-1.82050900	2.84314900	-2.29658600
O	-0.95086600	3.04418700	-1.39758900
O	-2.70000100	1.98492100	-2.38118800
F	-2.58117300	3.64849100	-4.42432200
F	-0.49472900	3.91723000	-3.96162900
F	-1.98545600	5.11709700	-2.96353000

Sub5-TS2-PdOTf-OTf

Pd	0.11574400	0.43729800	0.24275900
C	-1.64206500	1.40495800	0.79233400
C	-3.12772700	1.98202100	2.60487600
C	-4.16539600	1.28437900	1.98811000
C	-3.95912600	0.61924000	0.78114200
C	-2.70193000	0.67388500	0.20057300
H	-3.30333200	2.48071800	3.55642400
H	-5.14637800	1.24971400	2.45931900
H	-4.75635800	0.05191300	0.30503600
C	-2.31811500	-0.04766200	-1.01507200
C	-1.86631400	2.03836300	2.01952100
H	-1.05054800	2.57622700	2.50098600
O	-1.11937600	-0.20632400	-1.30044600
O	-3.27011600	-0.54914500	-1.75751300
C	-2.82268000	-1.17558000	-2.96925600
H	-2.13616100	-1.99574400	-2.73557200
H	-3.72648400	-1.53961400	-3.45969800
H	-2.31284600	-0.42824200	-3.58672600
H	-1.03084700	2.19331200	-0.06289900
S	1.21795600	0.61939300	3.09185400
O	1.20057400	1.31965800	1.73025600
O	0.51720400	-0.65586900	3.08008700

C	3.00568700	0.21934400	3.22555500
F	3.39971000	-0.51489400	2.20243100
F	3.21616800	-0.45345300	4.35273100
F	3.71959900	1.33756600	3.25750700
O	0.96063900	1.56575900	4.16908700
S	1.33781800	-2.30231000	-0.55378400
O	1.85947500	-2.71895300	-1.84807600
O	-0.03587800	-2.65730300	-0.20786400
C	2.37371800	-3.19654200	0.67035100
F	2.25936800	-4.50674600	0.45735800
F	1.98192100	-2.93401400	1.90468700
F	3.65039900	-2.85809500	0.53755600
O	1.69410900	-0.86180600	-0.21621600
C	-0.11610700	2.76677700	-3.36825400
S	-1.46512000	3.28452100	-2.23812600
O	-1.81804200	4.62681900	-2.66636800
O	-0.75976100	3.25491400	-0.89638200
O	-2.47106000	2.22643100	-2.37022600
F	-0.58483100	2.70843400	-4.61216200
F	0.88223000	3.63657700	-3.32797400
F	0.33873700	1.56850900	-3.03137600

Sub6

C	-3.59515700	0.92467300	0.26830400
C	-3.10624500	0.76602200	-1.02715000
C	-2.12146200	-0.17763600	-1.28489200
C	-1.61369100	-0.96951800	-0.25335700
C	-2.10835000	-0.80840400	1.03831900
C	-3.09730600	0.13603000	1.29905100
H	-4.36520700	1.66678600	0.47246300
H	-3.49484200	1.38197800	-1.83647800
H	-1.72332500	-0.29504800	-2.29390600
H	-1.69720300	-1.41170200	1.84762600
H	-3.47318100	0.26014900	2.31313400
C	-0.51761200	-1.95641300	-0.53696100
H	-0.78535500	-2.59747600	-1.38720400
H	-0.34718800	-2.60873400	0.33067100
N	0.73456000	-1.29022300	-0.94539000
H	1.30838000	-1.77563600	-1.63067900
S	1.71574400	-0.61430400	0.20560400
C	1.39548900	1.18759100	-0.02219300
O	3.08862300	-0.81379100	-0.21771100
O	1.23323500	-0.93687000	1.53428000
F	1.66684200	1.52925300	-1.26946800
F	2.19394100	1.84375900	0.80191600
F	0.13868600	1.47491400	0.25568000

Sub7

C	-3.53501000	-1.15861200	-0.00002000
C	-2.15721600	-1.32022000	-0.00014000
C	-1.31007600	-0.20682900	-0.00010300
C	-1.86154200	1.07792300	0.00003500
C	-3.24486000	1.22257000	0.00014600
C	-4.08938200	0.11772300	0.00012100
H	-4.17729500	-2.03784900	-0.00004600
H	-1.72740000	-2.32350400	-0.00027100
H	-1.20374400	1.93935800	0.00002300
H	-3.66580900	2.22700300	0.00025200
H	-5.16975800	0.24891800	0.00020300
N	0.07542200	-0.46161600	-0.00019000
H	0.32093900	-1.44239500	0.00008800
C	1.11283100	0.43675400	-0.00022500
O	0.95446500	1.64631800	-0.00039100
C	2.53149900	-0.15519800	0.00006300
C	2.60019100	-1.67978000	-0.00046300
H	2.13725900	-2.11936000	0.89571300
H	2.13752000	-2.11869400	-0.89710500
H	3.65159300	-1.99713200	-0.00040200
C	3.23251500	0.38250100	-1.24954000
H	3.18886600	1.47702300	-1.27324000
H	4.28621700	0.07039600	-1.25436400
H	2.76131900	0.00086100	-2.16644000
C	3.23163300	0.38166600	1.25051800
H	4.28529800	0.06943600	1.25600500
H	3.18812800	1.47618200	1.27485000
H	2.75969200	-0.00045400	2.16684400

DMSO

S	0.00000400	0.24723900	-0.44680600
O	-0.00087000	1.48522100	0.39606500
C	-1.34064400	-0.81587300	0.17977700
H	-2.28841000	-0.32212900	-0.05606900
H	-1.30498800	-1.80045800	-0.30094600
H	-1.24014200	-0.90681200	1.26811200
C	1.34134600	-0.81454400	0.17986800
H	1.24044200	-0.90657400	1.26809400
H	1.30665400	-1.79877200	-0.30169700
H	2.28913600	-0.32034900	-0.05499300

NMP

C	1.77578900	0.51518100	-0.19832300
C	1.32796500	-0.90280800	0.14316100

C	-0.18377000	-0.87089000	-0.00562900
C	0.54841700	1.37103500	0.13051300
H	2.66872500	0.84037000	0.34591600
H	1.99494000	0.59040700	-1.27136100
H	1.55220800	-1.16745800	1.18701000
H	1.76153300	-1.68575300	-0.48720700
H	0.44291000	2.24090900	-0.53406400
H	0.57128200	1.75149000	1.16794600
O	-0.94166400	-1.81977100	-0.04800300
C	-1.92423500	0.87317600	-0.01120500
H	-2.14716300	1.57010000	-0.83122600
H	-2.55123200	-0.01846900	-0.11231200
H	-2.15978800	1.37215100	0.94152000
N	-0.55215700	0.45003600	-0.05332800

Sub6-intermolecular

C	3.70338100	2.07607200	1.36556500
C	3.32181600	0.77862700	1.02374500
C	2.09928900	0.55788900	0.40891300
C	1.25044800	1.65534800	0.10770400
C	1.63329800	2.94640300	0.51750600
C	2.86347300	3.15975300	1.12034800
H	4.66868100	2.23891600	1.84206200
H	3.97815900	-0.06060200	1.25468600
H	0.97931600	1.89622400	-1.17657400
H	0.95846800	3.77845100	0.31727200
H	3.16461800	4.16377900	1.41089200
N	0.33421500	-0.82661400	-0.61076400
Pd	-0.67249600	0.98282000	-0.10337600
O	-2.66023200	0.19782800	-0.32740100
O	-3.38893000	3.51651500	1.81631500
O	-1.44029800	2.77755300	0.49791700
C	-0.99783900	3.79422800	2.84676200
C	-3.96743900	-1.86578700	-1.15208000
O	-3.95368700	-1.12599900	1.36737700
H	-0.41968400	-1.47087800	-0.23068900
C	1.60925600	-0.83942700	0.16876800
H	2.36640500	-1.47002900	-0.31339200
H	1.34748900	-1.30862900	1.12785200
S	0.50243600	-1.07872300	-2.29700000
O	1.84591900	-0.69227200	-2.67752300
O	-0.67350700	-0.56410300	-2.96323300
C	0.42391300	-2.92985300	-2.41199400
F	1.35736700	-3.44071600	-1.62420200
F	0.66329200	-3.25496200	-3.66511500
F	-0.75918100	-3.35247100	-2.04070700
F	-3.19403400	-1.92531800	-2.23118100
F	-5.02560500	-1.12070400	-1.42113300
F	-4.35866900	-3.09041300	-0.83782900
F	-1.52744300	4.05532800	4.02849800
F	0.15396700	3.15164900	3.02331000
F	-0.74916200	4.93735700	2.21673000
S	-3.03359800	-1.14403900	0.25475300
S	-2.15795100	2.75549800	1.86682900
O	-1.84553400	-2.02913300	0.37069600
O	-2.09683100	1.44172700	2.48545900
C	0.53027100	2.22934700	-3.49486700
C	1.13128700	1.81638400	-4.81036800
C	0.02809000	2.09816300	-5.82786100
C	-1.24728400	2.08918700	-4.98390700
H	1.38322500	0.74922000	-4.72848700
H	2.07151500	2.34998800	-4.98593100
H	0.16932600	3.09000500	-6.27507500
H	-0.00827300	1.36841900	-6.64214500
H	-2.00170500	2.81257500	-5.31760900
H	-1.72116500	1.09694000	-4.93431700
O	1.20109700	2.32971600	-2.44012500
N	-0.77240100	2.45393400	-3.65096200
C	-1.70718000	2.81473900	-2.60747700
H	-2.27657500	1.93884700	-2.26570100
H	-1.17128600	3.24798700	-1.75853600
H	-2.40789400	3.56211200	-2.99853700

Sub6-intramolecular

C	3.99266400	1.46142900	0.89938200
C	3.60516400	0.20231200	0.44595200
C	2.27322900	-0.06142200	0.16123600
C	1.30631900	0.97320900	0.28794700
C	1.71200200	2.20887200	0.83552000
C	3.04689700	2.46004900	1.11425500
H	5.04257300	1.65369800	1.11285600
H	4.34663900	-0.59053200	0.34508500
H	0.53800900	1.44372900	-0.70380300
H	0.96805100	2.98743800	0.98621800
H	3.34913600	3.43034700	1.50159200
N	0.36702800	-1.55723300	-0.25981100
Pd	-0.57023800	0.14549600	0.56036600
O	-2.37844100	-0.78093500	1.10335100
O	-0.27231900	2.27907700	-1.45022000
O	-1.44764300	2.08361000	0.70789400

C	-1.52493800	4.39044200	-0.49163300
C	-3.79223900	-2.93136300	0.79780600
O	-2.81403200	-2.16607700	3.12966200
H	-0.10258000	-2.30646000	0.31410400
C	1.84583500	-1.47101000	-0.11122500
H	2.35596500	-1.89717300	-0.98378900
H	2.09586200	-2.10082400	0.75375900
S	-0.19325300	-1.63894400	-1.91349800
O	0.83753600	-1.03549400	-2.72845400
O	-1.58007200	-1.25284700	-1.93603200
C	-0.13443200	-3.47442500	-2.18921200
F	1.09099100	-3.90659300	-1.92767300
F	-0.43496900	-3.70031100	-3.44728000
F	-0.99455000	-4.05880000	-1.38899900
F	-3.47991300	-2.98151300	-0.48792400
F	-4.89885800	-2.23233200	0.95905200
F	-3.97446000	-4.16261600	1.24864000
F	-1.57260500	4.96660900	-1.67528600
F	-2.56147700	4.75439400	0.23306300
F	-0.40492900	4.74158800	0.12492400
S	-2.41881400	-2.15588600	1.73770100
S	-1.56816600	2.56401900	-0.72231200
O	-1.23514100	-2.96668300	1.36723100
O	-2.78087000	2.24801200	-1.43397800

Sub7-intermolecular

C	-3.38138700	1.51377400	1.05006900
C	-4.09378000	0.31802700	1.09315500
C	-3.59754000	-0.81267600	0.46133100
C	-2.37910700	-0.75952400	-0.21635400
C	-1.64290200	0.43917700	-0.29497200
C	-2.17741000	1.56234300	0.36493600
H	-3.76244400	2.39976900	1.55327700
H	-5.04041700	0.26014500	1.62718400
H	-4.15097600	-1.75143500	0.51069000
H	-1.27500100	0.74787800	-1.57181900
H	-1.62067500	2.49786400	0.32536400
N	-1.96405100	-1.96653200	-0.82531300
H	-2.68400100	-2.67691700	-0.88020300
C	-0.73597000	-2.35618300	-1.22811600
O	0.27472300	-1.64099000	-1.18166000
C	-0.54471700	-3.79159600	-1.70261400
Pd	0.44195600	0.28614300	-0.50494800
C	1.01873200	2.60612400	1.04480100
O	0.67464800	2.27412500	-0.15916400
O	1.21688900	1.90533200	2.00892000
C	2.94879200	-0.82401600	0.13406800
O	2.48409400	0.12199200	-0.60275300
C	4.47950200	-0.93183500	-0.03477500
C	1.07789800	4.14383100	1.14342900
O	2.36017100	-1.59878000	0.86469600
F	-0.15380400	4.64492700	0.91708400
F	1.47600100	4.55823400	2.33252500
F	1.88755000	4.66577300	0.21805200
F	4.77488800	-1.28410500	-1.29567100
F	5.00337000	-1.84510700	0.77339200
F	5.08242400	0.23313000	0.20944000
C	-1.84989500	-4.54413100	-1.94571200
H	-1.61804100	-5.54662800	-2.32676700
H	-2.42991300	-4.69540200	-1.02264800
H	-2.48401400	-4.05047400	-2.69632300
C	0.27510100	-3.76210000	-2.99412300
H	-0.27148400	-3.26019500	-3.80458300
H	1.22650900	-3.24172300	-2.84348400
H	0.48796200	-4.79048900	-3.31401200
C	0.25718200	-4.48361100	-0.59010100
H	1.18521700	-3.94073000	-0.37380000
H	-0.32340600	-4.54062000	0.34123000
H	0.50433900	-5.50766700	-0.90087000
S	-0.60361200	2.06372300	-3.61045900
C	-0.81121200	3.55800300	-2.61851100
H	-0.31156500	4.37968400	-3.14489100
H	-1.88533600	3.76002800	-2.55694300
H	-0.37007900	3.41719200	-1.62181000
C	1.17084800	1.81617500	-3.34837500
H	1.68998200	2.50147200	-4.02964700
H	1.46083700	2.00428100	-2.30812400
H	1.40240100	0.78097900	-3.62055400
O	-1.37736700	0.93675300	-2.87678600

Sub7-intermolecular-2

C	1.68679800	-0.12399600	4.24670200
C	2.25268100	1.15420700	4.31575500
C	2.31314800	1.96512200	3.18355200
C	1.79188900	1.49707600	1.97517600
C	1.21948700	0.20580000	1.86710700
C	1.17989400	-0.58506400	3.03738200
H	1.64914600	-0.75055000	5.13284300
H	2.64896300	1.52486100	5.25700200
H	2.74819300	2.95983400	3.24372600

H	1.60584400	-0.34818000	0.81691100
H	0.74886300	-1.57719000	2.96315100
C	1.14257000	2.38778300	-0.25296600
O	0.04472700	1.79109000	-0.34139700
Pd	-0.53027700	0.09375300	0.62896600
N	1.94031100	2.31971700	0.83228500
H	2.75746800	2.91490800	0.82290600
C	1.57721200	3.24553900	-1.44795400
C	1.69082900	2.27513400	-2.65149500
H	0.71671100	1.83516500	-2.88002700
H	2.03939500	2.82794900	-3.53084100
H	2.39011600	1.45703600	-2.44998600
C	0.46879200	4.28962600	-1.71130000
H	0.38580300	5.00267800	-0.88254600
H	0.70535300	4.85208000	-2.62100900
H	-0.50075700	3.80355100	-1.84324900
C	2.92445000	3.95792600	-1.22912600
H	2.89266700	4.66498200	-0.38918500
H	3.74789900	3.24932400	-1.07697300
H	3.16823100	4.54303700	-2.12128700
C	-4.47427000	0.71327400	-0.92773500
C	-3.18586000	0.90292100	-0.08357100
O	-2.27806000	0.04963200	-0.45137500
O	-3.13723500	1.75427100	0.78525000
F	-5.43804300	1.56563400	-0.56218200
F	-4.95261700	-0.54500900	-0.78182400
F	-4.22504200	0.90233200	-2.24114900
C	3.07722500	-2.15534900	-2.05607600
C	2.28398500	-1.85163800	-0.75254200
O	2.43375400	-0.64984700	-0.35317300
O	1.62896800	-2.77099800	-0.25124500
F	2.59789000	-3.24045700	-2.68832700
F	4.37815200	-2.38332300	-1.77283900
F	3.02743900	-1.12325000	-2.92893500
S	-1.91393700	-2.78084800	0.69207600
O	-1.06349300	-1.70198400	1.47326000
C	-1.43443900	-2.80069100	-1.06679700
H	-1.94047000	-3.66503100	-1.51035500
H	-1.81185700	-1.87582200	-1.50322900
H	-0.34714400	-2.88662000	-1.14171400
C	-1.02882000	-4.28735100	1.19861800
H	-1.47165800	-5.14748200	0.68792100
H	0.02376700	-4.15504800	0.93421400
H	-1.15572200	-4.38913800	2.27845000

Sub7-intermolecular-3

C	1.27909900	-3.93936100	-0.99404300
C	0.35142300	-4.17325600	-2.01135000
C	-0.70835800	-3.30358200	-2.22148600
C	-0.85584900	-2.17642000	-1.41275900
C	0.04981600	-1.92636500	-0.35161300
C	1.12882000	-2.82787100	-0.18733100
H	2.10874600	-4.62528700	-0.83927700
H	0.45935500	-5.04305100	-2.65733600
H	-1.41588000	-3.48468200	-3.03076000
H	-0.49478500	-1.65613400	0.73130100
H	1.84089200	-2.62612500	0.61081800
C	-2.21865800	-0.10214700	-1.26195000
O	-1.34287700	0.65328900	-0.79567400
Pd	0.48426200	0.10883600	-0.02744700
N	-1.97163900	-1.36604500	-1.66976600
H	-2.77638200	-1.86250600	-2.03484700
C	-3.58203100	0.49632800	-1.58845800
C	-3.98151900	1.47307300	-0.48223000
H	-3.20224200	2.22218200	-0.30420800
H	-4.90331100	1.99129300	-0.77882400
H	-4.16147800	0.93657600	0.45593200
C	-3.39614600	1.25248500	-2.91276800
H	-3.09257600	0.57900500	-3.72718600
H	-4.34503900	1.72470800	-3.20010700
H	-2.63637600	2.03894200	-2.81710700
C	-4.67738600	-0.55678500	-1.74269000
H	-4.54553700	-1.18626900	-2.63717000
H	-4.74123500	-1.18670500	-0.84710100
H	-5.64180200	-0.04917600	-1.87252800
C	4.60393500	-0.51591700	0.70050100
C	3.30804100	0.06473300	0.09575000
O	2.29143800	-0.37210700	0.73909600
O	3.36535400	0.80818800	-0.87315000
F	5.68042500	-0.10364800	0.04338900
F	4.56948100	-1.85356100	0.64299600
F	4.73480100	-0.16327700	1.97865800
C	-2.73953200	0.12064000	2.90589700
C	-2.28716700	-0.92168700	1.86304400
O	-1.08951500	-1.29631600	2.02578400
O	-3.07328400	-1.18788800	0.95044300
F	-4.06879200	0.21119400	2.98718600
F	-2.28108900	1.31941400	2.50290000
F	-2.26789600	-0.11064500	4.12568200

S	1.25904900	2.85325700	-1.13350300
O	0.91700000	2.16782000	0.20850000
C	0.07340000	4.21648300	-1.15112000
H	0.27185100	4.88309100	-1.99789700
H	-0.91775600	3.76180300	-1.24900400
H	0.13601300	4.75438300	-0.19838700
C	2.73945100	3.80768300	-0.75466000
H	2.93187800	4.51937800	-1.56524500
H	2.58126200	4.32313400	0.19949000
H	3.55369300	3.08176900	-0.67727100

H	1.13206900	-4.21134000	-0.15948300
H	-0.46235500	-4.99430600	-0.03353700
C	0.76890000	-2.92745000	-2.54562800
H	1.61681600	-2.54083200	-1.96600900
H	1.12885700	-3.72729800	-3.20630700
H	0.37878600	-2.11402800	-3.17454600
C	-1.48052100	-4.03990500	-2.43399000
H	-2.29357100	-4.42318900	-1.79956800
H	-1.88693400	-3.29874600	-3.13822800
H	-1.12479800	-4.88291900	-3.03926200

Sub7-intramolecular

C	-3.32840200	1.20157100	1.79482200
C	-4.12176500	0.11404200	1.43571700
C	-3.65088100	-0.83658400	0.53783800
C	-2.38154700	-0.69529600	-0.01480800
C	-1.58159500	0.42958900	0.27462500
C	-2.07666700	1.35003400	1.21699600
H	-3.69426500	1.93448000	2.51042700
H	-5.11109500	-0.00810200	1.87246500
H	-4.25335700	-1.71273000	0.29860700
H	-1.12771800	1.15358900	-0.74164300
H	-1.46740600	2.22064200	1.46104500
N	-1.91215200	-1.73295700	-0.86086000
H	-2.59503300	-2.19953600	-1.44684800
C	-0.70226400	-2.31980600	-0.72702700
O	0.14383700	-1.90422100	0.08840000
C	-0.31960500	-3.48764200	-1.61448400
Pd	0.50991300	0.12108900	0.12068400
C	0.24112200	2.68904300	-0.96848100
O	0.97242100	2.12296100	-0.12204900
O	-0.80292400	2.23936500	-1.50016700
C	2.90448400	-1.03825600	0.91701300
O	2.46910100	-0.40182300	-0.11433200
C	4.16574100	-1.84941500	0.56127400
C	0.62591300	4.11966700	-1.38030500
O	2.42212300	-1.11346500	2.03026400
F	0.67871100	4.20633000	-2.70453000
F	-0.31408900	4.95563600	-0.93832600
F	1.79107800	4.48768800	-0.87951000
F	5.06587200	-1.12757700	-0.10311800
F	3.80222200	-2.87578800	-0.23144300
F	4.75057600	-2.35686700	1.63714000
C	0.27922700	-4.58794200	-0.73454400
H	0.62794500	-5.40904100	-1.37422600
